# Multifunctional hybrid films made from CoT_3_OT_x4_ and CoFeT_2_OT_4_ nanoparticles inside a poly 3-hydroxybutyrate matrix and study of their impact in methyl orange photodegradation

**DOI:** 10.1371/journal.pone.0312611

**Published:** 2024-10-31

**Authors:** Lan J. Bernal-Sánchez, América R. Vázquez-Olmos, Roberto Y. Sato-Berrú, Esther Mata-Zamora, Margarita Rivera, Vicente Garibay-Febles

**Affiliations:** 1 Instituto de Ciencias Aplicadas y Tecnología, Universidad Nacional Autónoma de México, Mexico City, Ciudad de México, México; 2 Instituto de Física, Universidad Nacional Autónoma de México, Mexico City, Ciudad de México, México; 3 Instituto Mexicano del Petróleo, Mexico City, Ciudad de México, México; National Research Centre, EGYPT

## Abstract

This work aims to produce hybrid materials with potential applications in dye photodegradation. Therefore, hybrid films were obtained by incorporating cobalt (II, III) oxide (Co_3_O_4_) or cobalt ferrite (CoFe_2_O_4_) nanoparticles (NPs) with 18 ± 1.6 nm and 26 ± 1.3 nm, respectively, into a poly 3-hydroxybutyrate (P3HB) polymeric matrix. The Co_3_O_4_@P3HB and CoFe_2_O_4_@P3HB hybrid films were fabricated by solvent casting in a ratio of 85 mg to 15 mg (P3HB-NPs). Different spectroscopic and microscopy techniques characterized the Co_3_O_4_ and CoFe_2_O_4_ NPs and the P3HB, Co_3_O_4_@P3HB and CoFe_2_O_4_@P3HB films. The optical band gap for Co_3_O_4_ and CoFe_2_O_4_ NPs was estimated from their diffuse reflectance spectra (DRS) around 2.5 eV. X-ray diffraction (XRD) of the hybrid films revealed that the nanometric sizes of the Co_3_O_4_ and CoFe_2_O_4_ nanoparticles incorporated into the P3HB are preserved. The magnetic hysteresis curve of CoFe_2_O_4_ nanoparticles and CoFe_2_O_4_@P3HB film showed a ferromagnetic behaviour at 300 K. Transmission electron microscopy (TEM) confirmed the formation of nanocrystals, and scanning electron microscopy (SEM) provided evidence for the successful incorporation of the NPs into the P3HB matrix. The surface roughness and hydrophilicity of the hybrid films are increased compared to the P3HB film. The impact of the nanoparticles and the hybrid films on the photodegradation of methyl orange (MO) in its acidic form was studied. The photodegradation tests were carried out by direct sunlight exposure. The CoFe_2_O_4_@P3HB hybrid film achieved 85% photodegradation efficiency of a methyl orange solution of 20 ppm after 15 minutes of exposure to sunlight. After 30 minutes of exposure to sunlight, the nanoparticles and the hybrid films reached about 90% of the MO degradation. The results suggest that combining nanoparticles with the polymer significantly enhances photodegradation compared to isolated nanoparticles.

## Introduction

Over the past three decades, extensive research has been conducted to obtain nanostructured materials of several kinds. The primary chemical and physical synthesis methods produce nanostructured powders or colloidal dispersions [1–3]. However, in many cases, the applications of these nanomaterials are enhanced when dispersed in an organic or inorganic matrix that does not interfere with their properties or even improve them [4]. In this sense, several attempts have been made to integrate inorganic NPs into a polymeric matrix, forming hybrid materials (NPs@polymer) that interact through molecular or nanometric scale interactions [5,6]. These compounds have emerged as a promising alternative to exploring and improving the applications of metallic oxide nanoparticles due to their cost-effectiveness, photoconductivity of the electrons, low density, and other outstanding properties [4,6,7]. In this work, multifunctional hybrid materials were prepared by incorporating cobalt (II, III) oxide (Co_3_O_4_) and cobalt ferrite (CoFe_2_O_4_) nanoparticles into a poly 3-hydroxybutyrate (P3HB) matrix to evaluate their potential in dye photodegradation. The Co_3_O_4_ NPs are an antiferromagnetic p-type semiconductor with a narrow optical band gap that varies from the range of 1.45 to 2.7 eV [8]. This compound is a mixed-valence oxide [Co(II)Co(III)_2_O_4_] with a normal spinel crystal structure based on an array of cubic close-packed oxide ions, in which Co(II) ions occupy the tetrahedral 8a sites, and Co(III) ions occupy the octahedral 16d sites [9]. This cobalt oxide has many industry applications, including biomedical technology [10], anode materials for rechargeable Li-ion batteries [11], energy device storage [12], and especially as an effective catalyst [13]. Otherwise, CoFe_2_O_4_ is also a semiconductor with a narrow bandgap ranging from 0.11 to 2.6 eV [14]. This ferrite has an inverse spinel-type structure, where the tetrahedral positions are occupied by trivalent cations (B). In contrast, the divalent anions (A) and trivalent cations (B) occupy the octahedral positions in a spatial arrangement [B]_Td_[AB]_oh_O_4_ [15]. The particle size, morphology and surface effects of said ferrite determine its applications in different areas [16], which are related to magnetic, magneto-optic, electrical, chemical, electrochemical, thermal, photoelectrochemical, thermoacoustic, and adsorption properties [17], including its use as a catalyst for removing pollutants in an aqueous medium [18–23].

On the other hand, biopolymers offer a promising alternative to conventional polymers, which are linked to fossil resource consumption, microplastic formation, non-degradability, and limited end-of-life options. Among these biopolymers, poly (3-hydroxybutyrate) (P3HB) emerges as a promising option [24]. This is a thermoplastic and isotactic polymer with a tensile strength like polyethylene. It is insoluble in water and highly crystalline, with a glass transition temperature of 4°C, a melting temperature of 180°C, and a degradation temperature of around 270°C [25]. Due to these properties, it has potential applications in several fields, such as biodegradable packaging [26,27], controlled drug release [28], and synthetic prostheses [29]. Therefore, combining P3HB with metal oxide nanoparticles can create hybrid films, incorporating the nanoparticles’ chemical and physical properties and the polymer’s mechanical properties [30]. Despite the extensive research on metallic oxide NPs and biopolymers, limited studies exist on creating multifunctional hybrid materials employing these molecules [31–38]. Moreover, it has been reported that around 280,000 tons of synthetic dyes are released annually through industrial discharge [39]. These dyes are resistant to biodegradation and can harm aquatic life and photosynthesis. Among these dyes, Methyl orange (MO) is widely used in the cosmetic, pharmaceutical, and textile industries. It is also known to cause skin diseases, respiratory tract infections, and eye irritation [39]. Therefore, removing and decolorizing these harmful dyes from water resources is crucial. Even though the degradation of MO has been studied extensively, conventional wastewater treatment plants still need to find ways to eliminate this molecule, considering its effects on the environment and human health [40–42]. This study represents the first successful endeavor to produce hybrid films using Co_3_O_4_ and CoFe_2_O_4_ NPs and the poly (3-hydroxybutyrate) (P3HB) polymer. The NPs and hybrid films obtained were also investigated for their effectiveness in the photodegradation of MO in its acid form.

## Materials and methods

### Materials

All chemical reagents were purchased from Sigma-Aldrich and were used as received without further purification. Cobalt (II) chloride hexahydrate (CoCl_2_•6H_2_O) (purity = 98%), iron (III) chloride hexahydrate (FeCl_3_•6H_2_O) (purity≥98%), sodium hydroxide (NaOH) (purity≥98%), and acetone (CO(CH_3_)_2_) (99.5%). Poly (R)- 3-hydroxybutyric acid (P3HB) and anhydrous chloroform (purity≥99%). Methyl Orange ACS reagent and linseed oil.

### Nanoparticles synthesis

The Co_3_O_4_ NPs were obtained from 1x10^-3^ moles (0.24 g) of cobalt (II) chloride hexahydrate (CoCl_2_•6H_2_O) grounded in an agate mortar with 2x10^-3^ moles (0.08 g) of sodium hydroxide previously milled. The mixture was milled for 30 minutes until the powder remained unchanged. The obtained product was a dark green Co(OH)_2_ powder, which was washed four times with cold distilled water and then twice with acetone. In each case, the product was separated by centrifugation at 3500 rpm for 10 minutes. Finally, it was air-dried at room temperature and heated to 600°C for 2 hours. The resulting product was a black powder made up of Co_3_O_4_ nanoparticles. The general chemical reaction is as follows:

$$3CoCl_{2}\cdot 6H_{2}O+6NaOH+\frac{1}{2}O_{2}\overset{OO}{\underset{\begin{array}{c} 600^{{}^{\circ}}C/2hrs\\ \Delta \end{array}}{\to }}Co_{3}O_{4}(NPs)+6NaCl+21H_{2}O$$

**Fig 1:**
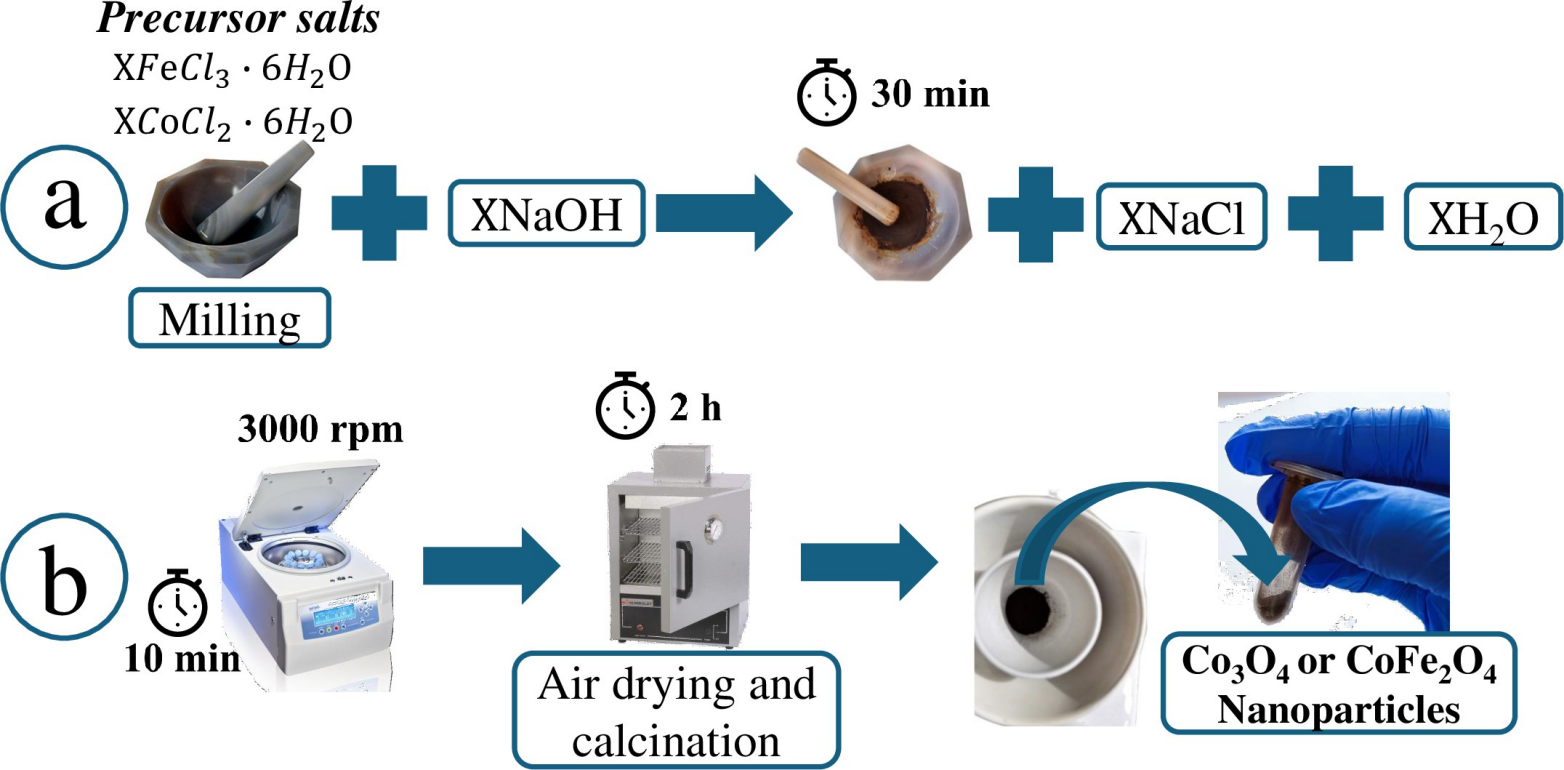
General procedure for obtaining Co_3_O_4_ and CoFe_2_O_4_ nanoparticles. (a) Milling the precursor metallic salts with sodium hydroxide for 30 minutes. (b) The product is washed, air-dried, and then calcinated for 2 hours.

Furthermore, CoFe_2_O_4_ NPs were synthesized following the previous procedure from 1x10^-3^ moles (0.24 g) of cobalt (II) chloride hexahydrate (CoCl_2_•6H_2_O) and 2x10^-3^ moles (0.54 g) of iron (III) chloride hexahydrate (FeCl_3_•6H_2_O) which was milled with 1x10^-3^ moles (0.32 g) of sodium hydroxide (NaOH). A dark brown powder was obtained, washed, air-dried, and then heated at 800°C for 2 hours. Finally, a black powder formed by CoFe_2_O_4_ NPs was obtained. The general chemical reaction is presented below, and the general procedure to get the nanoparticles is depicted in Fig 1.


$$CoCl_{2}\cdot 6H_{2}O+2FeCl_{3}\cdot 6H_{2}O+8NaOH\overset{O}{\underset{800^{{}^{\circ}}C/2hrs}{\to }}CoFe_{2}O_{4}(NPs)+8NaCl+22H_{2}O$$

### Hybrid film synthesis

**Fig 2:**
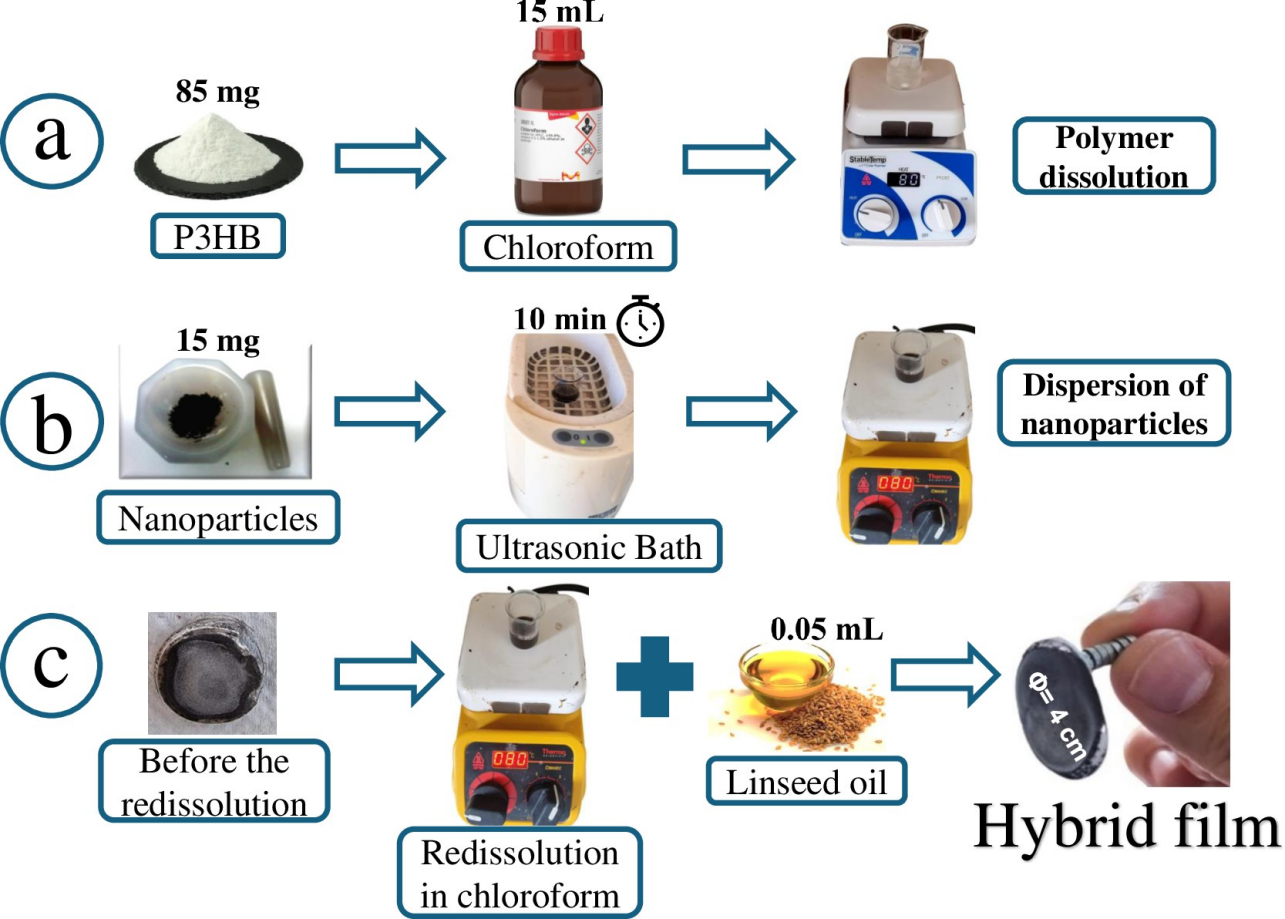
General procedure for obtaining the NPs@P3HB hybrid films. (a) P3HB is dissolved in chloroform. (b) The NPs are dispersed in chloroform and added to the P3HB solution. (c) Hybrid film forms after the first film is redissolved.

The hybrid films were obtained by solvent-casting method (Fig 2). First, 85 mg of the finely poly (3-hydroxybutyrate) (P3HB) was dissolved in 15 mL of chloroform at 80°C under constant stirring for 10 minutes (Fig 2A). Then, in an ultrasonic bath, 15 mg of the previously synthesized Co_3_O_4_ or CoFe_2_O_4_ NPs were dispersed in 10 mL of chloroform for 10 minutes (Fig 2B). After dissolving the P3HB and dispersing the NPs, both phases were mixed in a beaker at 80°C while constantly stirring. A film was formed when around 90% of the solvent evaporated. This film was dissolved in 25 mL of chloroform and added 0.05 mL of linseed oil, which acts as a plasticizer. The mixture was heated at 80°C and constantly stirred to produce the final hybrid film (Fig 2C).

#### Instruments for nanoparticles and hybrid films characterization

The X-ray diffraction patterns were performed with Cu Kα radiation (λ = 1.5406 Å) in a diffractometer Empyrean PIXel 1D Malvern Panalytical; diffraction intensity was measured between 10° and 70°, with 2θ step of 0.01°, for 0.25 s per point. The average crystal size *(D)* of the NPs was estimated from their diffractograms by the Debye-Scherrer formula, *D* = Kλ/βcosθ where *K* is the shape factor equal to 0.9, λ is the Cu Kα radiation, β is the full width at half maximum intensity of selected peaks (FWHM), and θ is the Bragg angle. Transmission electron micrographs were obtained with a (TEM) FEI Tecnai F20 microscope, operating at 200 KV S/TEM with an X-TWIN lens and a high brightness field emission electron gun (FEG). Raman spectra of 100 to 900 cm^−1^ were acquired in a Nicolet Almega XR dispersive Raman spectrometer and detected by a CCD camera at 25 s and a resolution of ~4 cm^−1^. The excitation beam was an Nd:YVO_4_ 532 nm laser and the incident power on the sample was ~3 mW. Furthermore, 1 mg of NPs with 100 mg of potassium bromide (KBr) was pressed into a pellet. The pellet was adjusted and analyzed with a Nicolet Nexus 670 Fourier Transformed Infrared (FTIR) spectroscopy from 4000 to 400 cm^-1^ with a resolution of 4 cm^-1^. Ultraviolet-visible (UV–Vis) absorption spectra of the powdered samples were obtained by the diffuse reflectance technique with an Ocean Optics USB2000 miniature fiber-optic spectrometer. The nanoparticles’ optical band gap energy was determined by Tauc’s plot, obtained from their corresponding Diffuse Reflectance Spectra (DRS). According to Tauc’s equation [43] for a direct bandgap:

$${\left(\alpha hv\right)}^{2}=A(hv-\text{Eg})$$ (1)

where *α*(2.303 cm^-1^) is the absorption coefficient, hυ is the photon energy, $hv=\left(\frac{1239}{\kappa }\right)(\text{eV}),$Eg the bandgap energy (eV), and A is constant depending on the type of transition. When *αh*υ = 0,Eg = hυ. The bandgap energy is determined by plotting (*αh*υ)^2^ versus *h*υ and finding the intercept on the *h*υ axis by extrapolating the plot to(*αh*υ)^2^ = 0. The magnetization curves of the CoFe_2_O_4_ NPs were obtained at room temperature in a Vibrating Sample Magnetometer (VSM) Quantum Desing MPMS3 with an applied field of 20 kOe.

Scanning Electron micrographs were obtained with Philips equipment ESEM XL 30 at 5 kV to characterize the films. Raman spectra of 200 to 3500 cm^−1^ were acquired with a WItec Alpha 300 XR dispersive Raman spectrometer and detected in a 180° backscatter configuration. The excitation beam was an Nd: YVO_4_ 532 nm laser and the incident power on the sample was ~1 mW. The topographic profiles and roughness parameters were performed in an Atomic Force VEECO NANOSCOPE IV+Multimode MMAFM/ST microscope. The tapping mode was employed with a scanning rate of 1 Hz and a scan resolution of 256 lines. The scan area was set at 50 μm x 50 μm. We used a Tap300GD-G 300 kHz, 40 N/m tip. All images were processed with the software NanoScope Analysis v1.40. Water drops (30 μL) were applied to three areas of the film’s surface with Pocket Goniometer Model PG-1 for contact angle measurement.

#### Photodegradation assays

**Fig 3:**
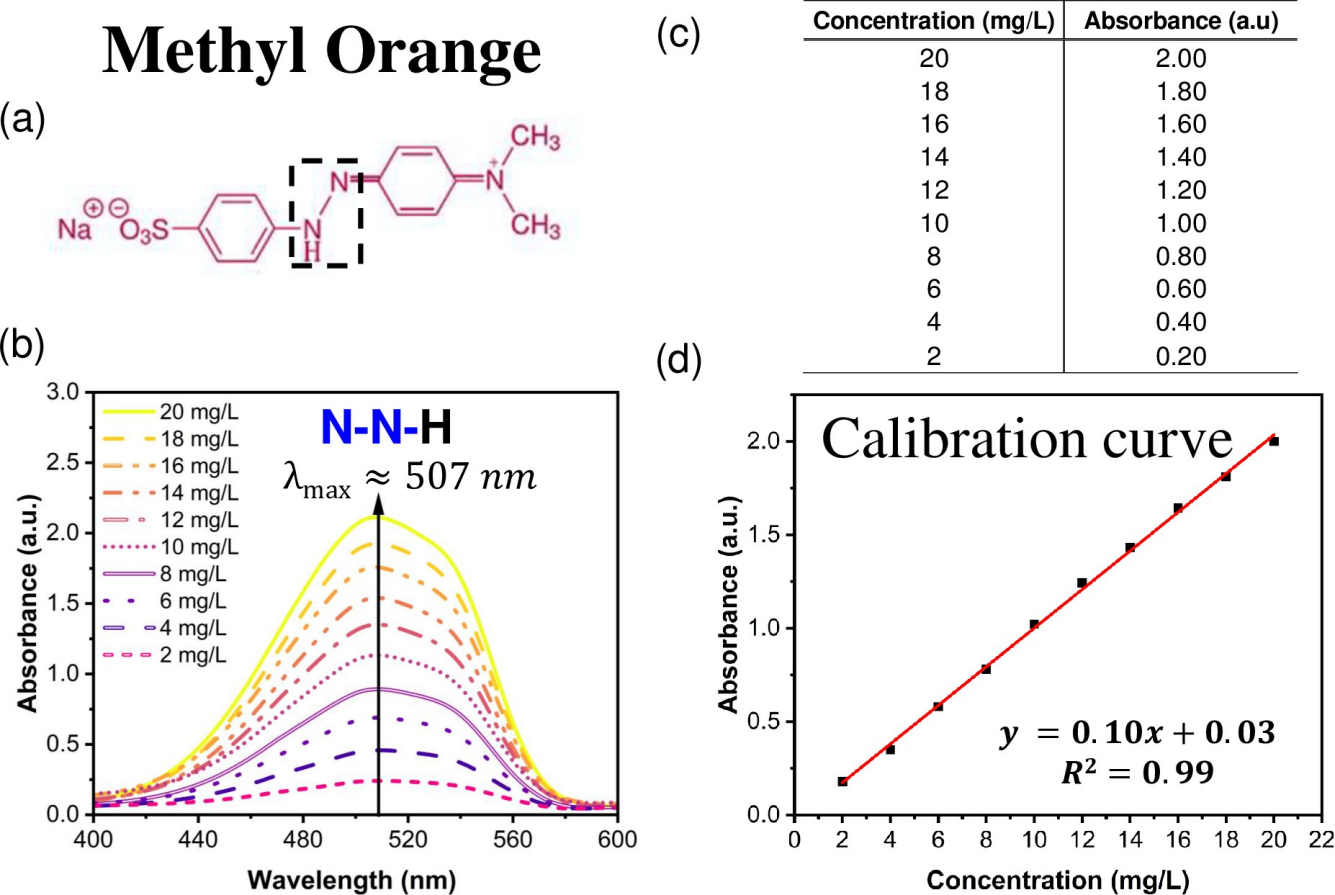
Photodegradation assays. (a) Methyl Orange (MO) structure in its acid form, (b) UV-Vis spectra of MO from 2 to 20 mg/L, (c) Table of the MO concentration *vs* absorbance and (d) calibration curve.

Methyl Orange solutions were prepared at pH 3 with concentrations ranging from 20 to 2 mg/L (20-2ppm). The UV-Vis spectra of these solutions were recorded, and the band at 507.40 nm corresponding to the quinoid structure (N-N-H) of the MO in its acid form was used as a reference to create a calibration curve, as shown in Fig 3.

A stock solution of MO (20 mg/L) was prepared in darkness and adjusted to pH 3.0 by adding 0.1M of hydrochloric acid (HCl). Then, 15 mg of Co_3_O_4_ or CoFe_2_O_4_ NPs were added to 30 mL of the MO solution, and the mixture was exposed to sunlight under constant stirring. For 2 hours, 1 mL of the mixture was collected every 5 minutes during the experiment. The same procedure was followed with the Co_3_O_4_@P3HB and CoFe_2_O_4_@P3HB hybrid films. Once the experiment was completed, the corresponding dispersions were stored in amber bottles to prevent direct exposure to light. All the experiments were repeated three times to ensure reproducibility. The percentage of dye photodegradation can be estimated using the following expression [44].


$$n=\left(\frac{C_{0}-C}{C_{0}}\right)X100(\%)$$ (2)

C_0_ (mg/L) is the dye concentration at the initial stage, and C (mg/L) is the dye concentration at specific time intervals upon sunlight illumination. Additionally, the films were subjected to a recycling process after using the hybrid films for the first time (cycle 1). Subsequently, they were washed three times with distilled water to remove the adsorbed dye on its surface. Then, it was kept in an oven at 60°C for 24 hours to eliminate water molecules and reused for the photodegradation of methyl orange. The same procedure was repeated three times. The kinetic model of the fabricated nanoparticles and hybrid films was thoroughly analyzed with an equation to study the relationship between time (*t*) in minutes as the *x*-axis and *In(C*_*0*_*/C)* as the y-axis [44]. The methyl orange photodegradation rate constant (k) (min^-1^) was calculated by Eq (3).


$$ln\left(\frac{C_{0}}{C}\right)=kt$$ (3)

#### Instruments for photodegradation assays

The intensity changes of the band corresponding to the (N-N-H) group for methyl orange in its acid form were recorded with a UV-Vis spectrophotometer (Ocean Optics, model USB2000).

## Results and discussion

### Characterization of the Co_3_O_4_ and CoFe_2_O_4_ nanoparticles

**Fig 4:**
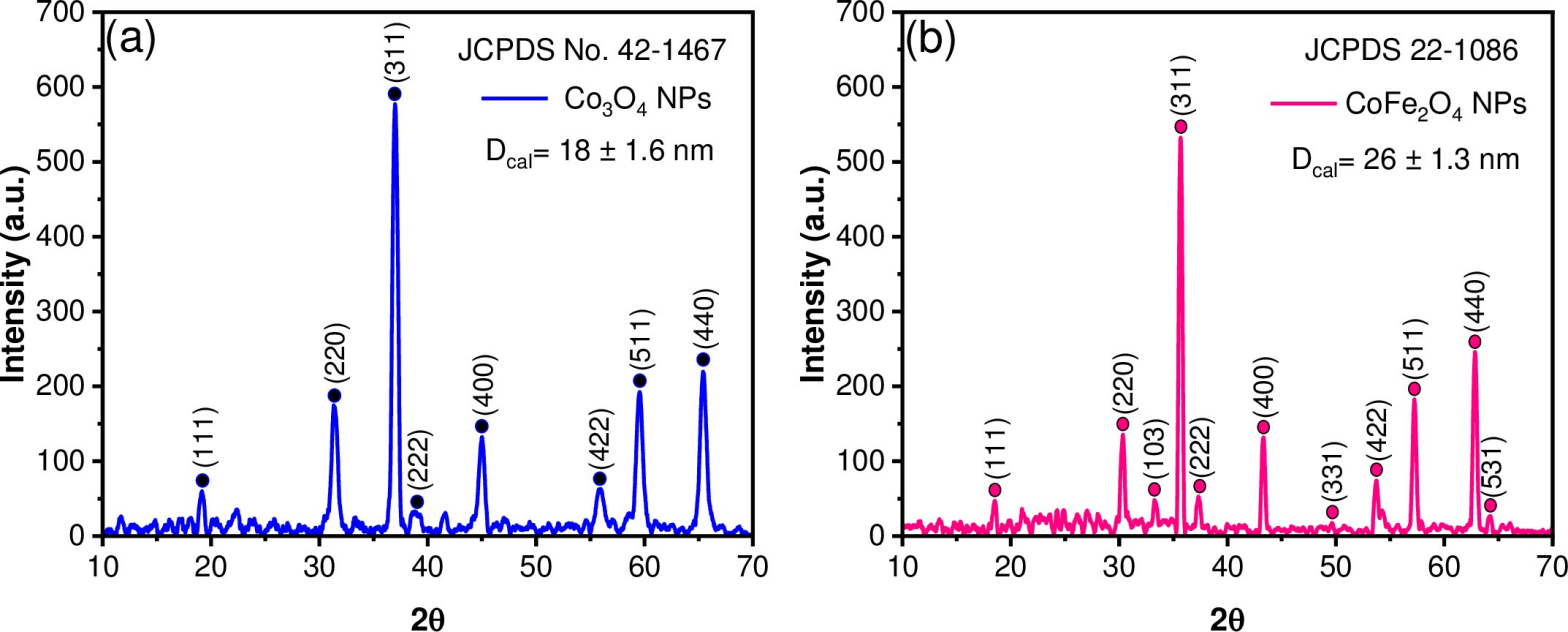
XRD patterns of the NPs. (a) Co_3_O_4_ and (b) CoFe_2_O_4_.

**Fig 5:**
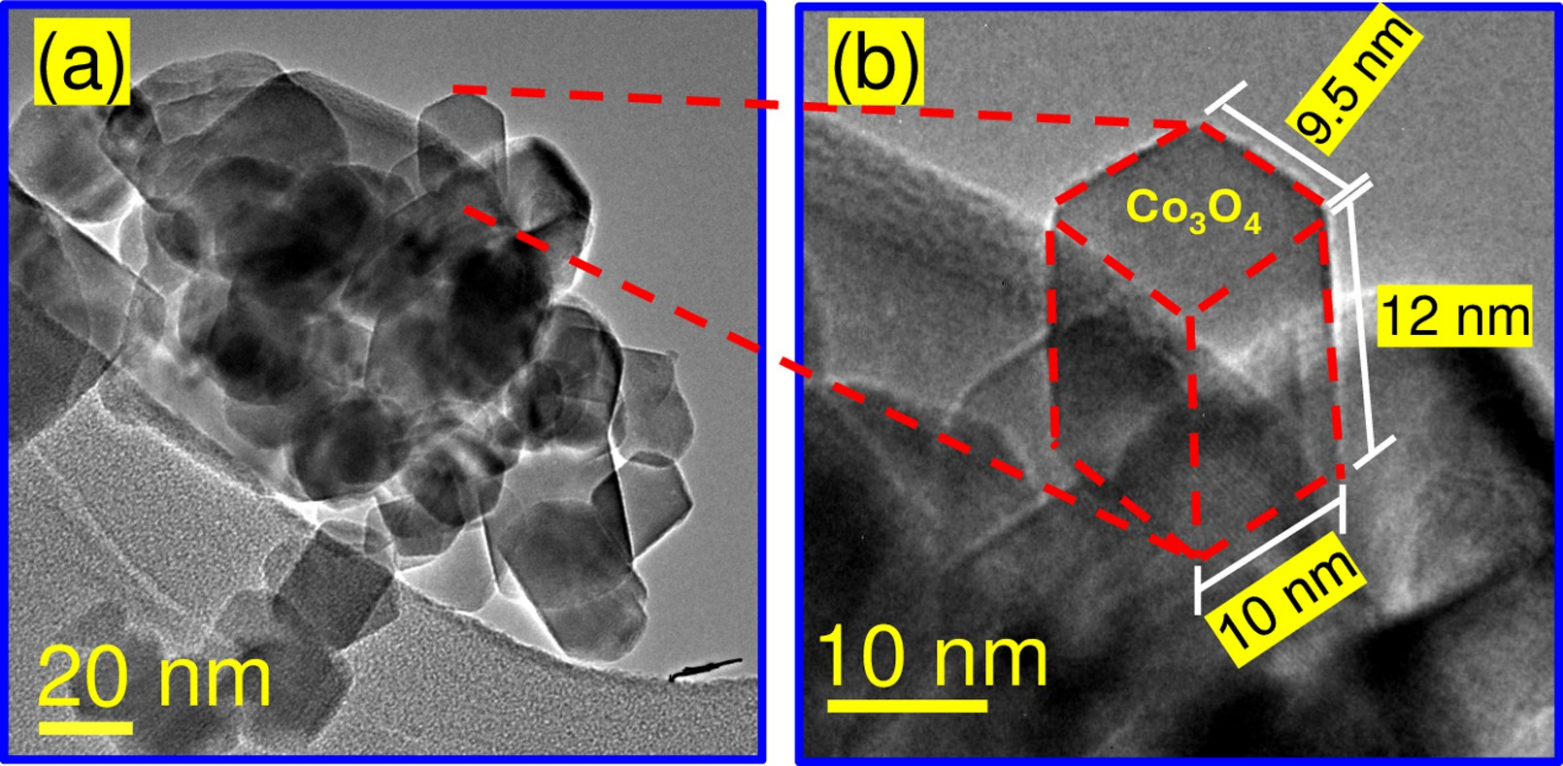
Representative TEM images of Co_3_O_4_ NPs. (a) Cuboid shapes and (b) zoom image of a cuboid.

**Fig 6:**
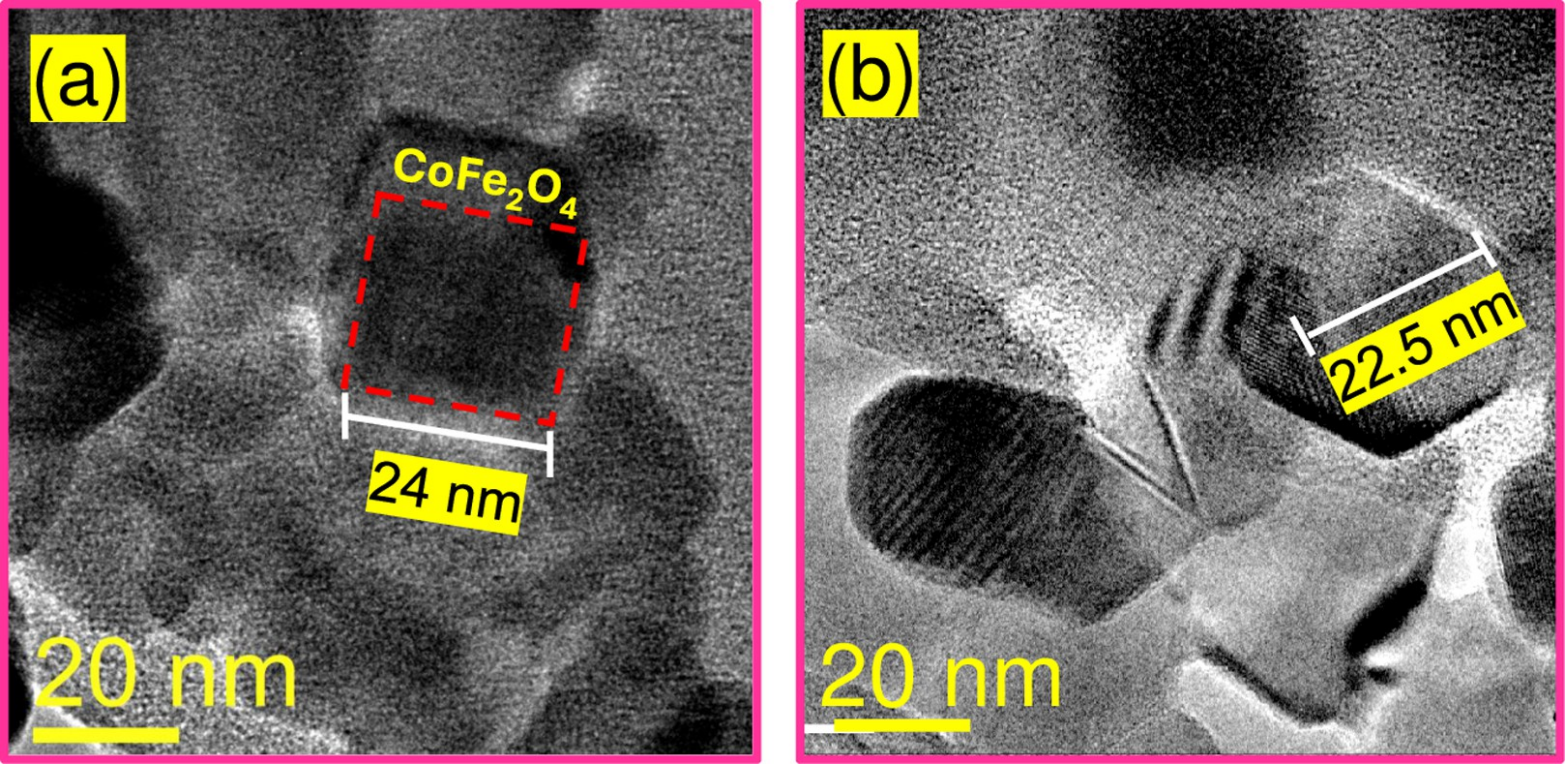
Representative TEM images of CoFe_2_O_4_ NPs. **(a)** Cube and **(b)** cuboid shapes.

The powder X-ray diffraction patterns of Co_3_O_4_ and CoFe_2_O_4_ NPs are shown in Fig 4. For cobalt (II, III) oxide NPs, all reflections in the XRD patterns **(**Fig 4A**)** can be attributed to the face-centered cubic structure, with space group Fd-3m and crystal lattice parameters a = 8.083 Å. According to the Joint Committee on Powder Diffraction Standards (JCPDS) 42–1467 card [45], the main characteristics peaks at 19.2° (111), 31.4° (220), 37.0° (311), 38.7° (222), 45.0° (400), 56.0° (422), 59.5° (511), 65.4° (440) are observed. On the other hand, the XRD pattern of the CoFe_2_O_4_ NPs **(**Fig 4B**)** corresponds to inverse spinel-type structure and cubic phase, with the same space group as de cobalt oxide, and crystal lattice parameters a = 8.4 Å according to the JCPDS 22–1086 card [46], in this case, all the reflections appear at 18.5° (111), 30.3° (220), 33.3° (103), 35.7° (311), 37.3° (222), 43.3° (400), 49.6° (331), 53.7° (422), 57.2° (511), 62.8° (440), 64.2° (531). In both systems, no additional peaks owning to other phases are observed. Scherrer’s equation estimated the average crystallite sizes of 18 ± 1.6 nm and 26 ± 1.3 nm for Co_3_O_4_ and CoFe_2_O_4_ NPs, respectively. Figs 5 and 6 show representative TEM micrographs of each oxide, confirming the presence of nanocrystals with close dimensions to those determined from their corresponding X-ray diffraction patterns. Representative images of Co_3_O_4_ particles with cuboid shapes are shown in Fig 5A. A zoom image of a cuboid measuring 12 nm in length, 10 nm in width, and 9.5 nm in height dimensions is depicted in Fig 5B. Moreover, Fig 6A and 6B show representative TEM images of a cube and a cuboid shape of CoFe_2_O_4_ measuring approximately 24 nm and 22.5 nm, respectively.

**Fig 7:**
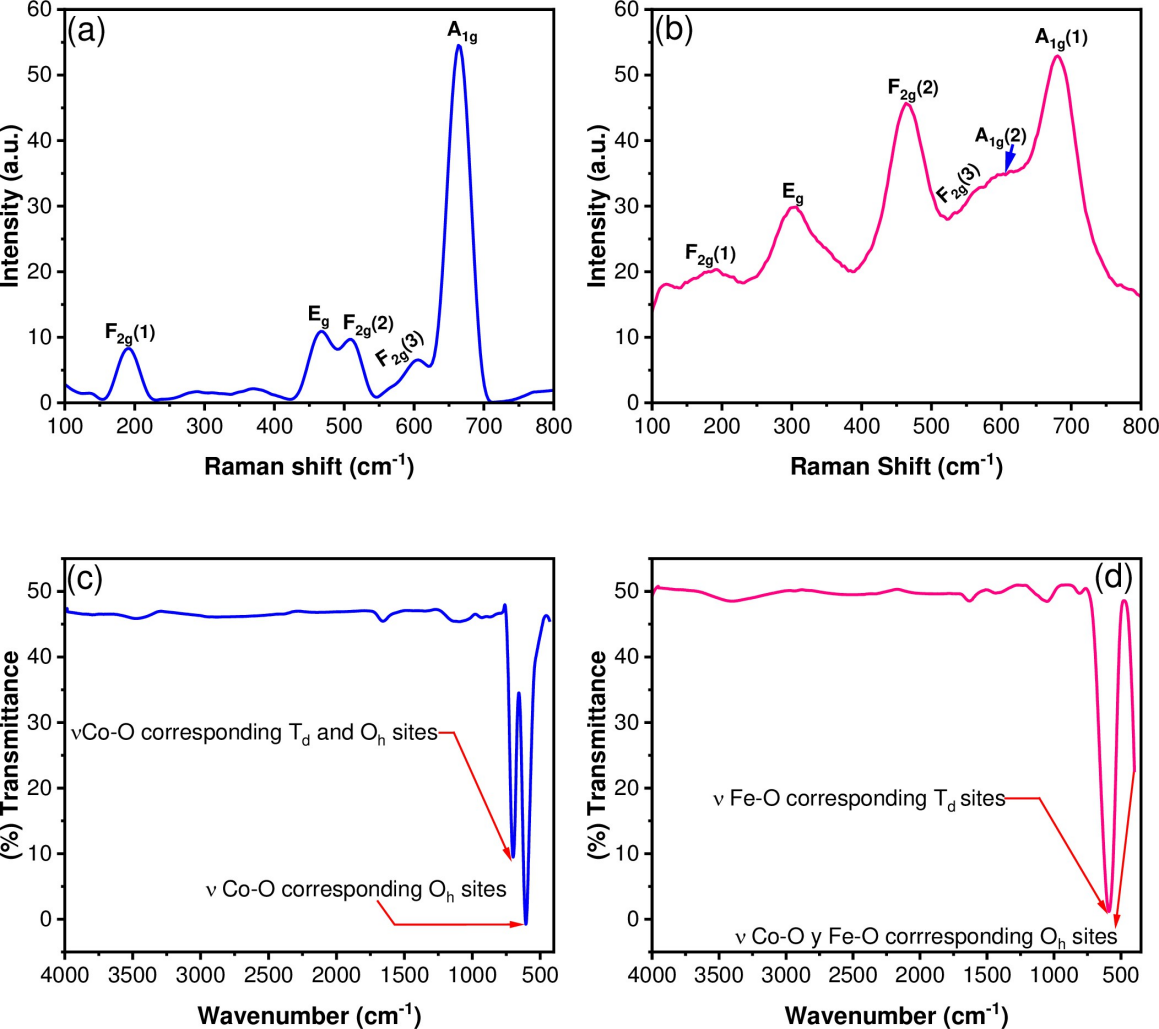
Raman spectra of (a) Co_3_O_4_ NPs and (b) CoFe_2_O_4_ NPs. FT-IR spectra of (c) Co_3_O_4_ NPs and (d) CoFe_2_O_4_ NPs.

On the other hand, Raman scattering spectra of the nanostructured powder of Co_3_O_4_ and CoFe_2_O_4_ NPs (Fig 7) confirms the structure and composition of the phases. Raman spectroscopy is an effective technique for identifying lattice phenomena exhibiting vibrational mode variations. For normal spinel $Co^{2+}{\left(Co^{3+}\right)}_{2}O_{4}^{2-}$ where *Co*^2+^ and *Co*^3+^ ions occupy the sites tetrahedral and octahedral, respectively. This compound presents a symmetry group$O_{h}^{7}$. According to group theory, the following irreducible representation is predicted:

$$\Gamma =A_{1g}+E_{g}+3F_{2g}+5F_{1g}+2A_{2u}+2E_{u}+2F_{2u}$$

Being five modes active in Raman$\left(A_{1g}+E_{g}+3F_{2g}+5F_{1g}\right)$. Raman spectrum of the Co_3_O_4_ NPs (Fig 7A) shows five characteristic bands in the 100 to 800 cm^-1^ regions. The signal with a maximum of 665 cm^-1^ was assigned to the *A*_*1g*_ mode, characteristic of the octahedral sites. In contrast, the signal at 468 cm^-1^ corresponding to the *E*_*g*_ mode and the signals with a maximum at 190, 508, and 605 cm^-1^ associated with the three modes *F*_*2g*_ are related to the combined vibrations of the tetrahedral site and octahedral oxygen motions [47]. Similar behavior has been observed for CoFe_2_O_4_ NPs (Fig 7B). In this case, six main signals are shown. The peak at 680 cm^-1^ (*A*_*1g*_ (1)) has been assigned to the symmetric stretching of Fe-O_4_ tetrahedral bonds, proving the inverse-spinel structure. The signals at 302 cm^-1^ (*E*_*g*_) and 560 cm^-1^ (*F*_*2g*_ (3)) are related to the symmetric and asymmetric bending of oxygen concerning Fe (or Co), respectively. The peak at 464 cm^-1^ (*F*_*2g*_ (2)) is assigned to the asymmetric stretching of the Fe(Co)-O bond, whereas the signal at 188 cm^-1^ (*F*_*2g*_ (1)) is attributable to the translational movement of the whole tetrahedron. Furthermore, an additional signal at 612 cm^-1^ (*A*_*1g*_ (2)) corresponds to the stretching vibrations of the Fe-O and M-O bonds in tetrahedral sites is observed [48]. Besides, the FT-IR spectra confirm the formation of the spinel structure for both the Co_3_O_4_ and CoFe_2_O_4_ nanoparticles. For cobalt oxide (Fig 7C), two characteristic signals are observed below 1000 cm^-1^ associated with *α*-*Co*_3_ *O*_4_spinel. The first band at 695 cm^-1^ was assigned to the bridging vibration of νCo-O mode at the octahedral and tetrahedral sites. In contrast, the peak at 604 cm^-1^ corresponds to the νCo-O stretching vibration mode at the octahedral sites. Also, a low-intensity band is observed around 3500 cm^-1^ due to water adsorption [49]. Besides, the spectrum for cobalt ferrite (Fig 7D) shows a band located at 589 cm^-1^ assigned to the νFe-O mode of the tetrahedral sites of the inverse spinel. Another signal at 399 cm^-1^ is associated with νCo-O and νFe-O vibrations in the octahedral sites [50].

**Fig 8:**
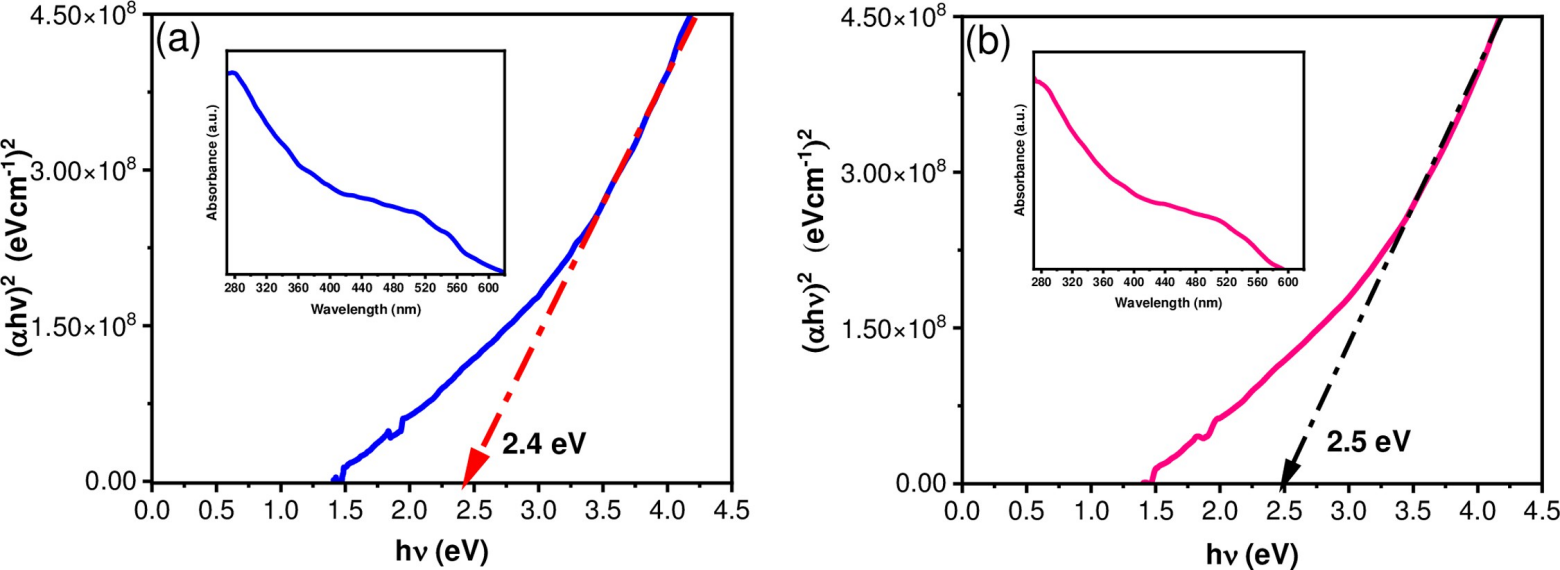
Optical band gap energy (Eg) obtained by Tauc’s plot method and UV-Vis DRS (inset). For (a) Co_3_O_4_ NPs and (b) CoFe_2_O_4_ NPs.

The Diffuse Reflectance Spectra (DRS) corresponding to Co_3_O_4_ and CoFe_2_O_4_ NPs are shown in Fig 8A and 8B, respectively. The sample’s optical band gap was determined by extrapolating the (αhv)^2^ (eVcm^-1^)^2^ versus *h*υ. The absorption energy corresponding to the band gap (Eg) at α = 0 is 2.4 eV for Co_3_O_4_ NPs and 2.5 eV for CoFe_2_O_4_ NPs when the value of *h*υ is extrapolated. A semiconductor metal oxide-mediated photodegradation involves exciting electrons from the valence band to the conduction band, generating electron-hole pairs. The electron and hole then interact with O_2_ and H_2_O to produce oxidative radicals that break down organic compounds. In this scenario, the Co_3_O_4_ and CoFe_2_O_4_ NPs have an energy band gap (Eg) that allows them to be active under visible light exposure [8,51].

**Fig 9:**
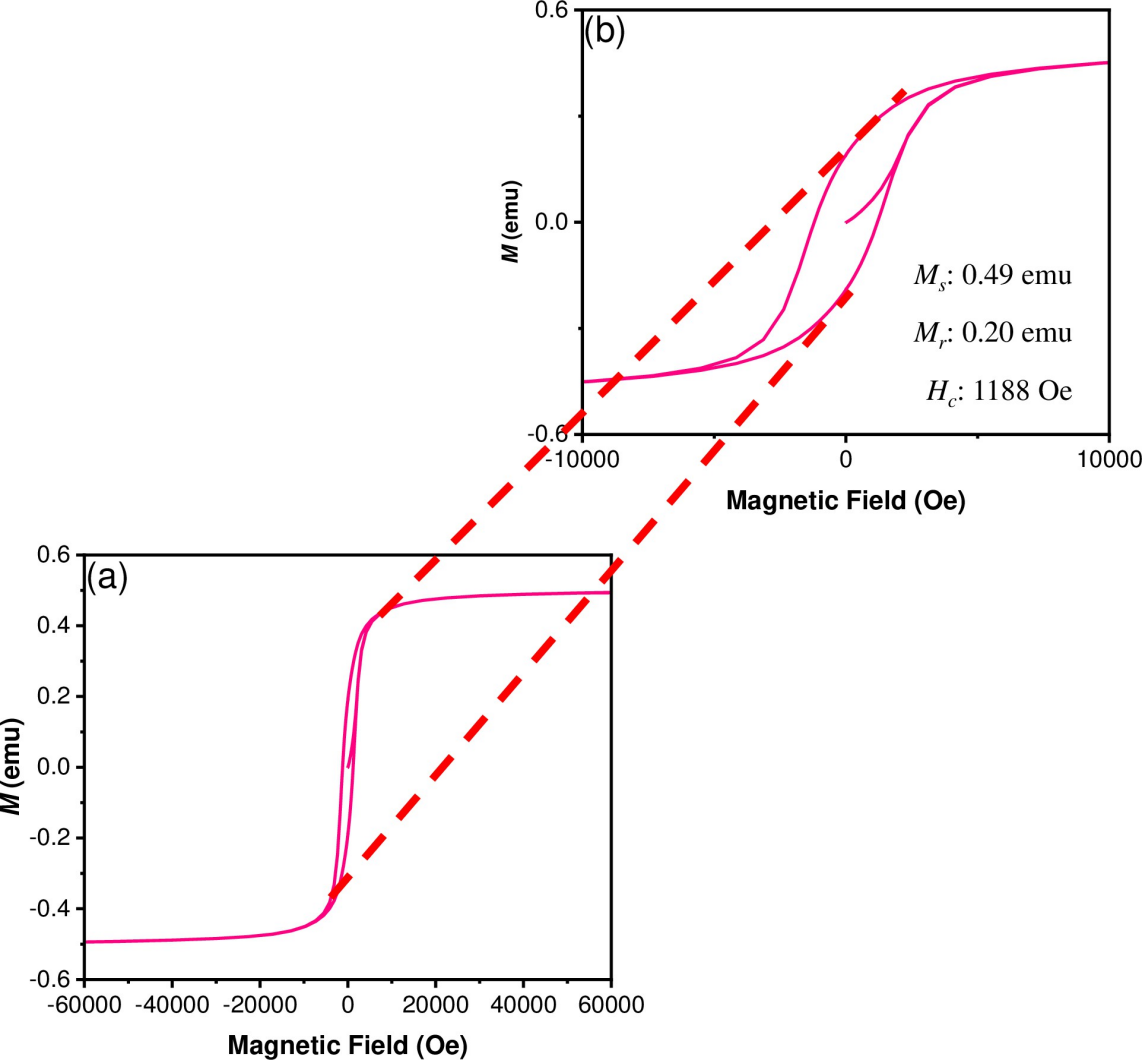
(a) Hysteresis curve (M-H) corresponding to CoFe_2_O_4_ NPs and (b) zoom image of the hysteresis loop.

**Fig 10:**
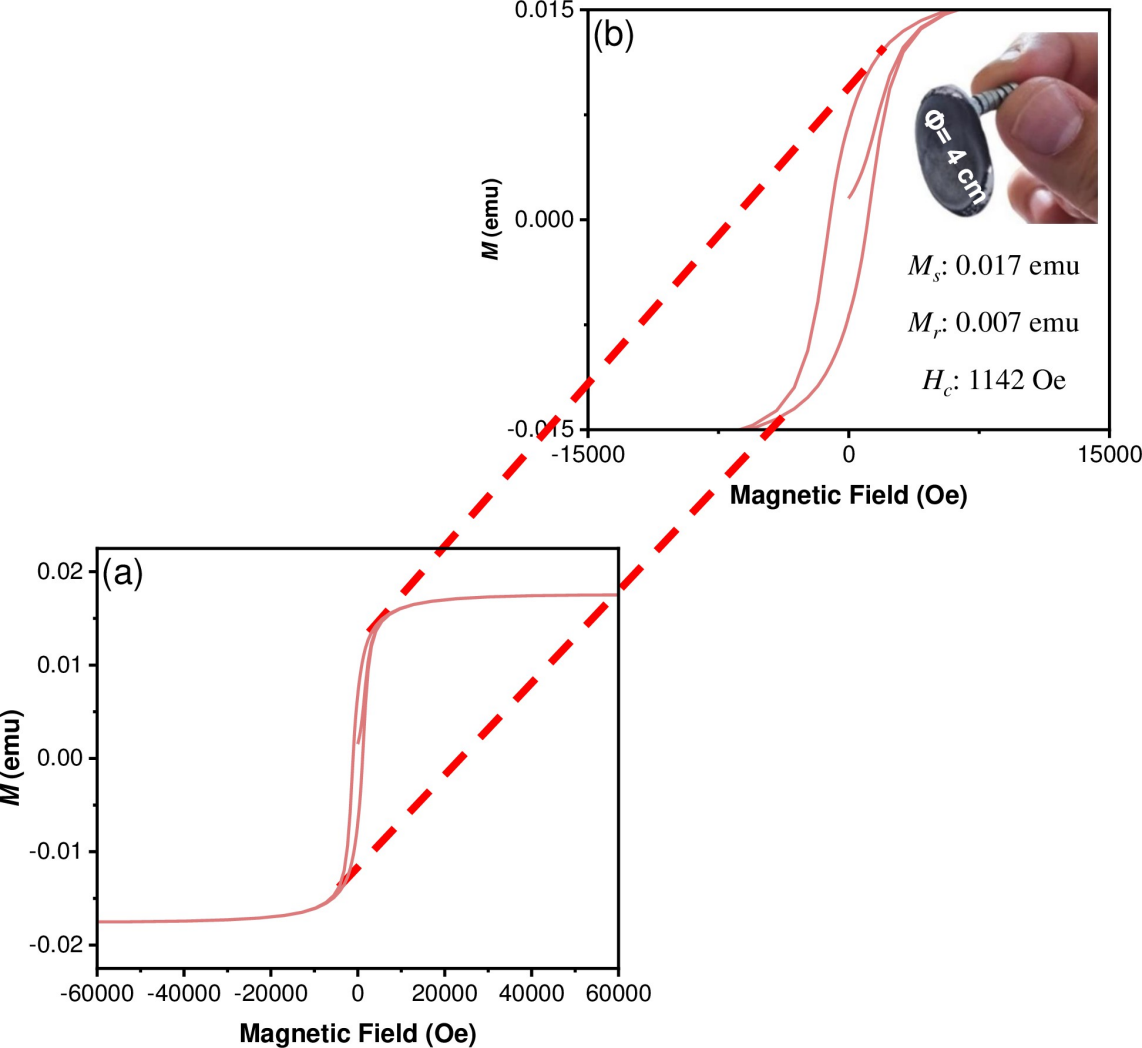
(a) Hysteresis curve (M-H) corresponding to CoFe_2_O_4_@P3HB hybrid film and (b) zoom image of the hysteresis loop, including an image of the hybrid film attracted by magnets.

Moreover, the hysteresis curve of the CoFe_2_O_4_ NPs **(**Fig 9A) was obtained from -60 to +60 KOe at 300 K. A characteristic ferrimagnetic behaviour was observed, with a saturation magnetization (Ms) of 0.49 emu, a remanence magnetization (Mr) of 0.20 emu and a coercive field (Hc) of 1188 Oe, as depicted in Fig 9B. The magnetization values observed are lower than those of cobalt ferrite in its bulk form and other nanostructures of these inverse spinel [51,52]. However, it is important to note that the magnetic properties depend not only on the size and shape of the nanoparticles but also on the synthesis method used in its preparation [52]. The hysteresis curve of the CoFe_2_O_4_@P3HB hybrid film (Fig 10A) was obtained under the same conditions used to get the CoFe_2_O_4_ NPs loop. A weak ferrimagnetic behavior was observed, with a Ms of 0.017 emu, a Mr of 0.007 emu, and a Hc of 1142 Oe. The magnetic response decreases in this case because the nanoparticles are incorporated into a diamagnetic polymer. Furthermore, once embedded in the polymer matrix, the distance between the nanoparticles increases, reducing their magnetic coupling. However, the magnetic coercivity is similar in both cases, indicating that the nanoparticles do not lose their magnetic force once inside the film. Although several magnetic parameters are reduced in the hybrid film compared to the isolated NPs, the CoFe_2_O_4_@P3HB film’s ability to magnetize is remarkable, as shown in Fig 10B.

### Characterization of the films

All films were characterized by Raman spectroscopy, X-ray diffraction (XRD), and scanning electron microscopy (SEM). The roughness and hydrophilicity of the films were evaluated by atomic force microscopy (AFM) and through contact angle measurement, respectively. The results obtained are presented below.

### Characterization of the P3HB film

**Fig 11:**
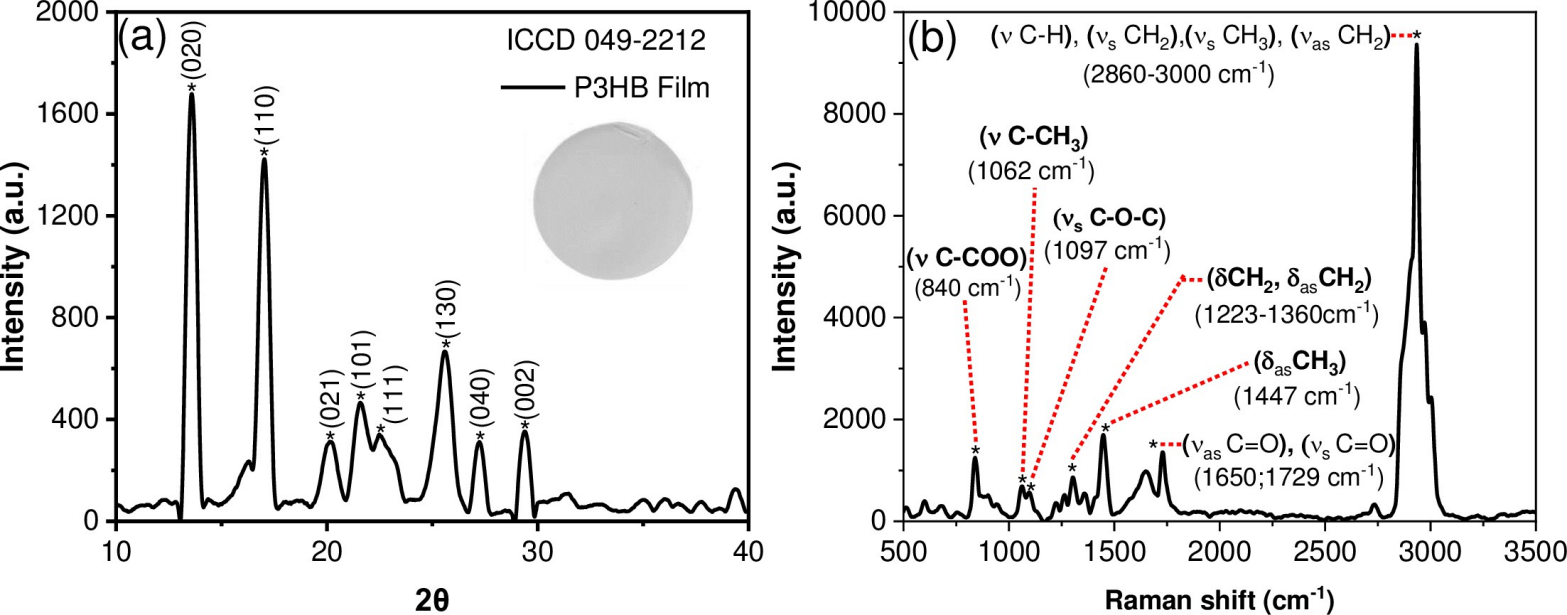
(a) X-ray diffraction pattern, including the P3HB film image and (b) its corresponding Raman spectrum.

The X-ray diffraction pattern of P3HB film is presented in Fig 11A. The diffraction peaks at 13.5°, 17.2°, 20.2°, 21.6°, 22.7°, 25.7°, 27.3°, and 28.5° were assigned to the (020), (110), (021), (101), (111), (130), (040) and (002) planes, respectively, according to those reported in the (International Center for Diffraction Data) crystallographic card ICDD 49–2212 and corresponds to those reported by Rincón-Granados et al. [37]. Also, the Raman spectrum of P3HB film (Fig 11B) was obtained, and the Raman active modes due to the biopolymer were observed. The thermal treatment during the film synthesis process produces the reorganization of the polymeric chains; therefore, the Raman signals in the range from 2860 to 3000 cm^-1^ corresponding to the group’s (νC-H), (ν_s_CH_2_), (ν_s_CH_3_), and (ν_as_CH_2_) appear slightly displaced. These bands are characteristic of the crystalline state of the polymer. The band at 1730 cm^-1^ is assigned to the vibration of the symmetric ν_s_C = O group, and the asymmetrical vibration of this group ν_as_C = O appears at 1650 cm^-1^. The band at 1447 cm^-1^ corresponds to the asymmetrical bending of methyl and methylene groups (δCH_2_, δCH_3_). Additionally, the bands between 1224–1360 cm^-1^ are associated with the bending of methylene groups (δCH), the wagging of methylene groups (*w*CH_2_) and the characteristic band of the helical configuration of the crystalline state. The band at 1097 cm^-1^ corresponds to the asymmetrical stretching vibration of the ester group (ν_as_C-O-C). The signal at 1062 cm^-1^ is identified as the vibration of the symmetric ν_s_C-CH_3_ group. The bands in the range from 870 cm^-1^ to 945 cm^-1^ are due to the stretching vibration of carbonated chains (νC-C), and the band at 840 cm^-1^ is a consequence of the stretching vibration of the νC-COO group. Finally, the bands between 200–800 cm^-1^ could be attributed to the out-of-plane bending of carbonyl groups (γC = O). These last bands are associated with the interactions between the polymer chains in their arrangement film.

### Characterization of the Co_3_O_4_@P3HB and CoFe_2_O_4_@P3HB hybrid films

**Fig 12:**
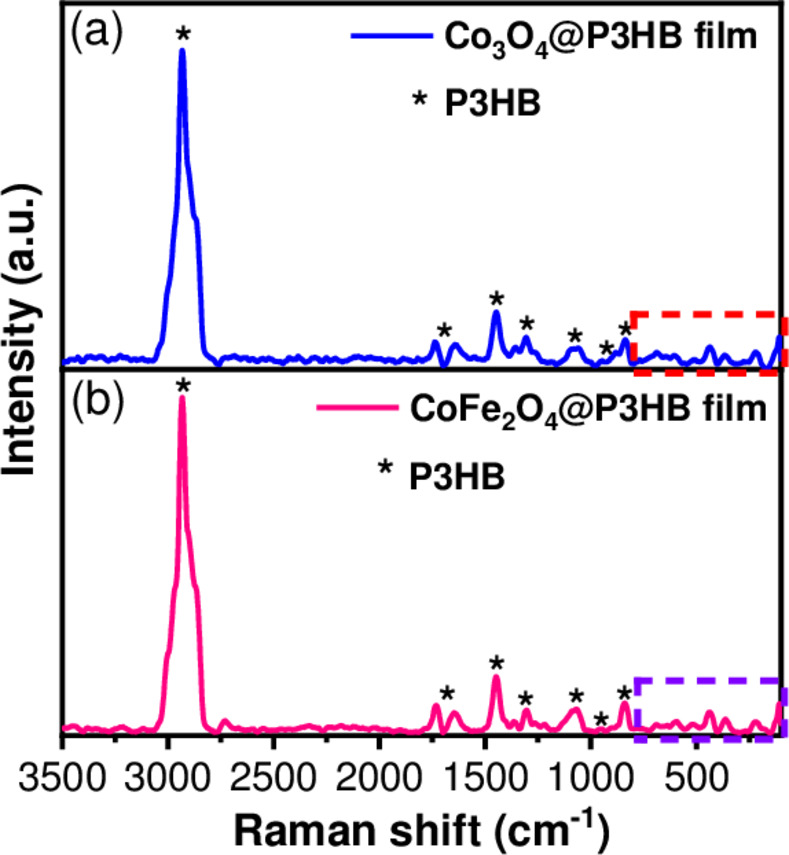
Raman spectra of (a) Co_3_O_4_@P3HB and (b) CoFe_2_O_4_@P3HB films.

The Co_3_O_4_@P3HB and CoFe_2_O_4_@P3HB hybrid films’ Raman spectra are shown in Fig 12A and 12B. There are not some noticeable changes when comparing the hybrid films to the P3HB film. The intensity of the polymer bands in the hybrid films remains unchanged because the amount of polymer is much higher than that of the NPs (15% NPs-85% P3HB w/w). However, the analysis of the hybrid films’ Raman spectra suggests that the interaction with the NPs occurs through the carbonyl and carboxyl groups of P3HB since the region from 840 to 200 cm^-1^ is the most affected in the hybrid films. Although there is evidence of interaction between the polymeric matrix and nanoparticles, XRD proved to be a more sensitive technique for analyzing these hybrid films.

**Fig 13:**
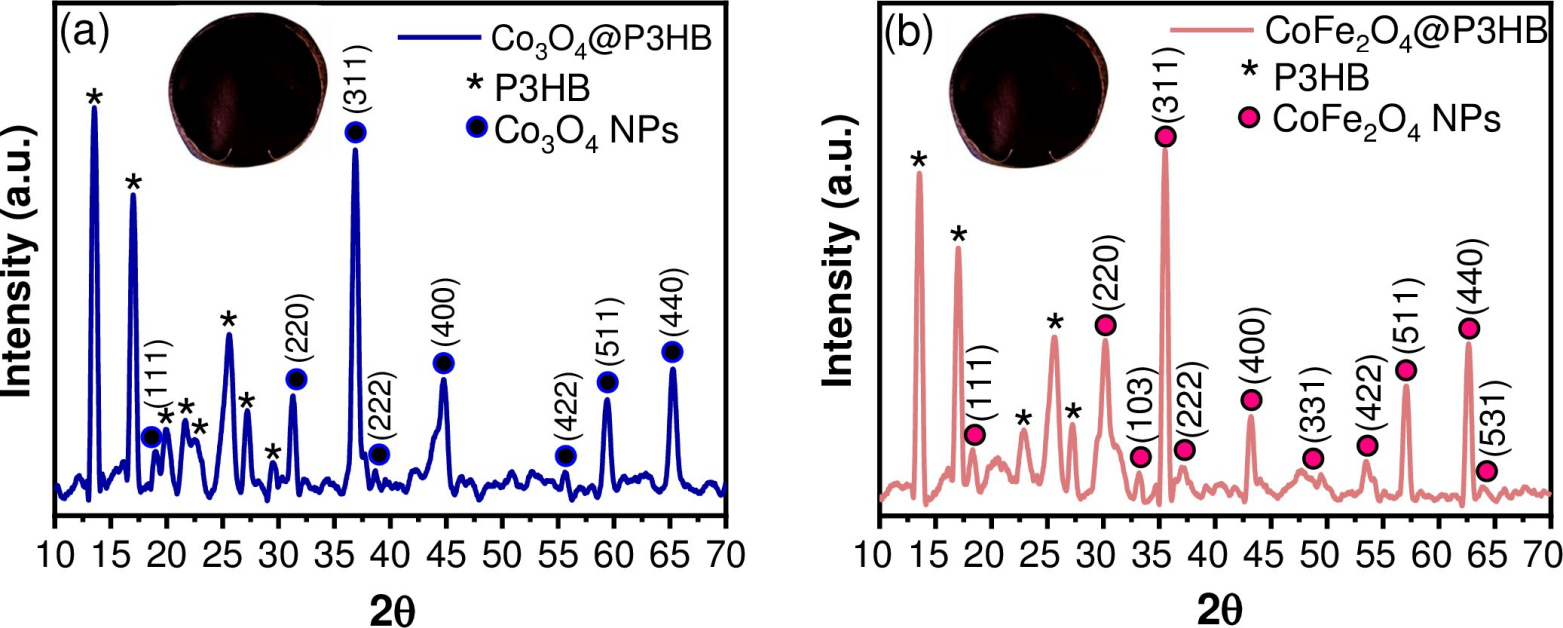
X-ray diffraction patterns of (a) Co_3_O_4_@P3HB and (b) CoFe_2_O_4_@P3HB. A representative image of the corresponding hybrid films is included.

Fig 13A and 13B show the XRD patterns of Co_3_O_4_@P3HB and CoFe_2_O_4_@P3HB hybrid films, respectively. The characteristic diffraction peaks of the P3HB and the Co_3_O_4_ (JCPDS 42–1467) or CoFe_2_O_4_ (JCPDS 22–1086 cards) are observed, which confirms that the NPs were successfully included in the polymeric matrix.

Furthermore, Scherrer’s equation estimated the average crystallite size of each oxide included in the hybrid film of 19 ± 1 nm for Co_3_O_4_@P3HB film (Fig 13A) and 27 ± 0.8 nm for CoFe_2_O_4_@P3HB film (Fig 13B). These sizes closely match those obtained from the XRD patterns of the crystallites of each oxide before being incorporated into the polymer matrix (18 ± 1.6 nm for Co_3_O_4_ and 26 ± 1.3 nm for CoFe_2_O_4_). Therefore, the experimental processing used to create the hybrid films did not affect the size of the NPs in the polymeric matrix.

**Fig 14:**
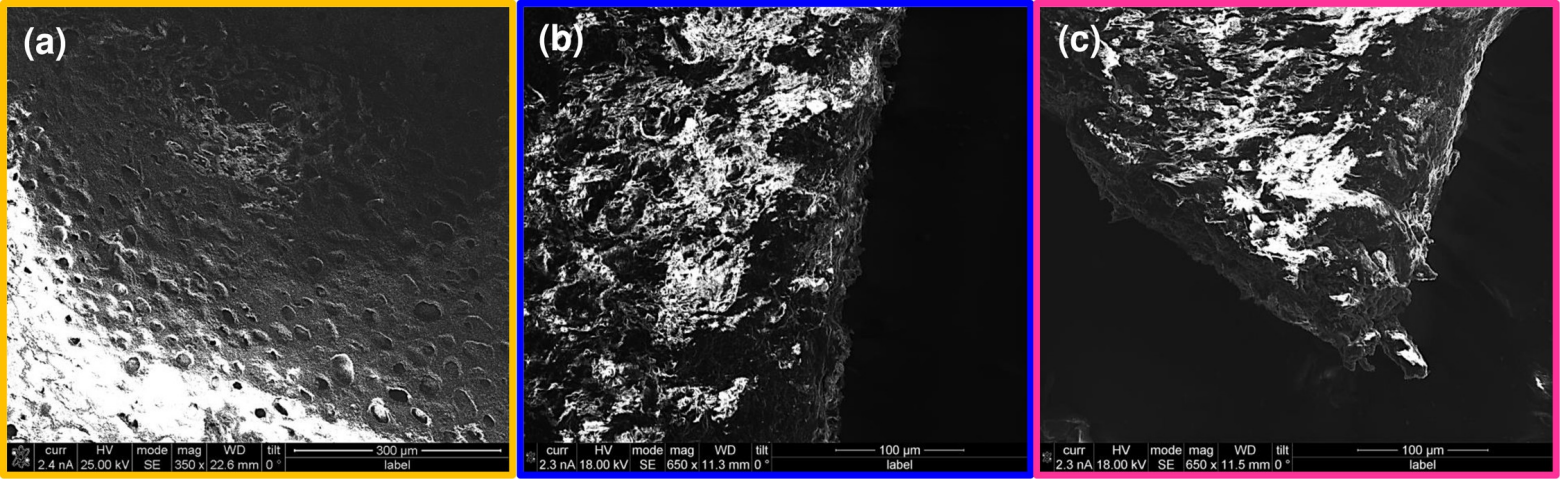
Representative SEM micrographs. (a) P3HB film, (b) Co_3_O_4_@P3HB, and (c) CoFe_2_O_4_@P3HB hybrid films.

On the other hand, SEM micrographs of the P3HB film and the NPs@P3HB hybrid films are presented in Fig 14. The first image (Fig 14A) shows the surface of P3HB films obtained by the solvent casting method. This surface has irregular pores of different sizes, possibly caused by the removal of the volatile solvent. Conversely, the surface of the hybrid films (Fig 14B and 14C) reveals a rough surface with no pores, suggesting that the presence of the nanoparticles provides a significant change in the surface. From a physicochemical point of view, slower kinetic solvent evaporation in the films containing the NPs could be the reason for the reductions in surface pore roughness.

The thickness of all the films was measured using a micro vernier at three different points on three films of each system studied. The P3HB film without NPs has a thickness of 551 ± 25 μm, while the Co_3_O_4_@P3HB and CoFe_2_O_4_@P3HB hybrid films have thicknesses of 261 ± 15 μm and 362 ± 19 μm, respectively. The method used to obtain the films is simple and demonstrates good reproducibility. The hybrid films are thinner than the P3HB film without NPs, partly because the latter has 15% less polymer by weight. In addition, the sizes of the NPs are different, with CoFe_2_O_4_ NPs being the largest (27 nm), which could also influence the thickness of the films.

**Fig 15:**
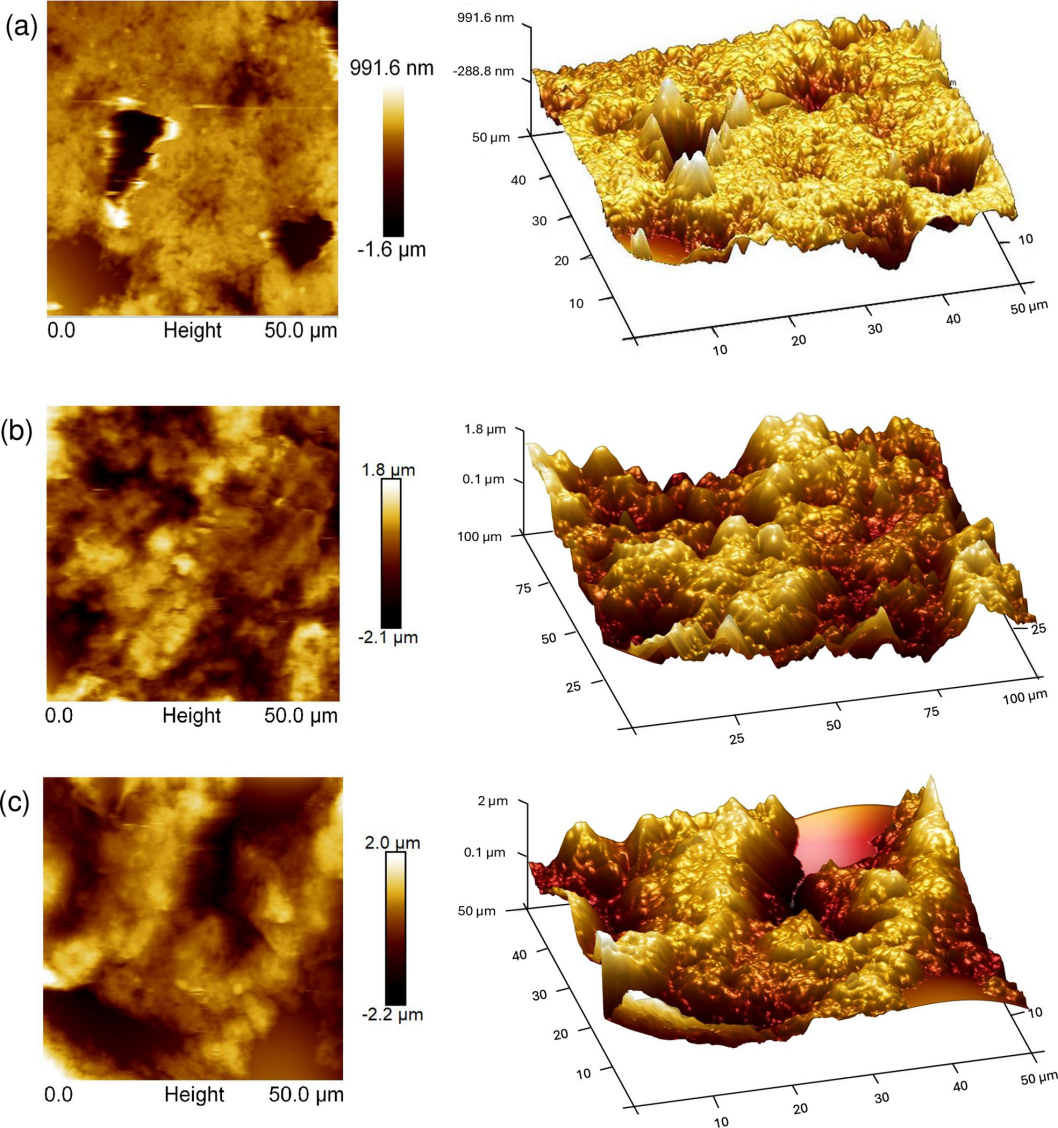
AFM height mapping 2D and 3D images. (a) P3HB film, (b) Co_3_O_4_@P3HB hybrid film, and (c) CoFe_2_O_4_@P3HB hybrid film.

On the other hand, the films’ surface roughness was analyzed using atomic force microscopy (AFM). Fig 15 shows the 2D and 3D images of the AFM height mapping, which illustrate the variations in surface topography between the hybrid films and the P3HB film. The root mean square (RMS) roughness was determined by averaging the measurements from three different lines on the surface of each sample. The RMS values were 18 ± 0.7 nm for P3HB, 26 ± 0.8 nm for Co_3_O_4_@P3HB, and 24 ± 0.6 nm for CoFe_2_O_4_@P3HB. These results suggest that adding Co_3_O_4_ or CoFe_2_O_4_ nanoparticles to a P3HB matrix results in a similar increase in the surface roughness of the hybrid films. The hybrid films show more consistent roughness across the surface, as observed in the 3D image and confirmed by the standard deviation values of the RMS measurements. Therefore, the roughness induced by nanoparticles in the P3HB matrix mainly occurs at the nanometer level, as expected for these hybrid materials.

**Fig 16:**
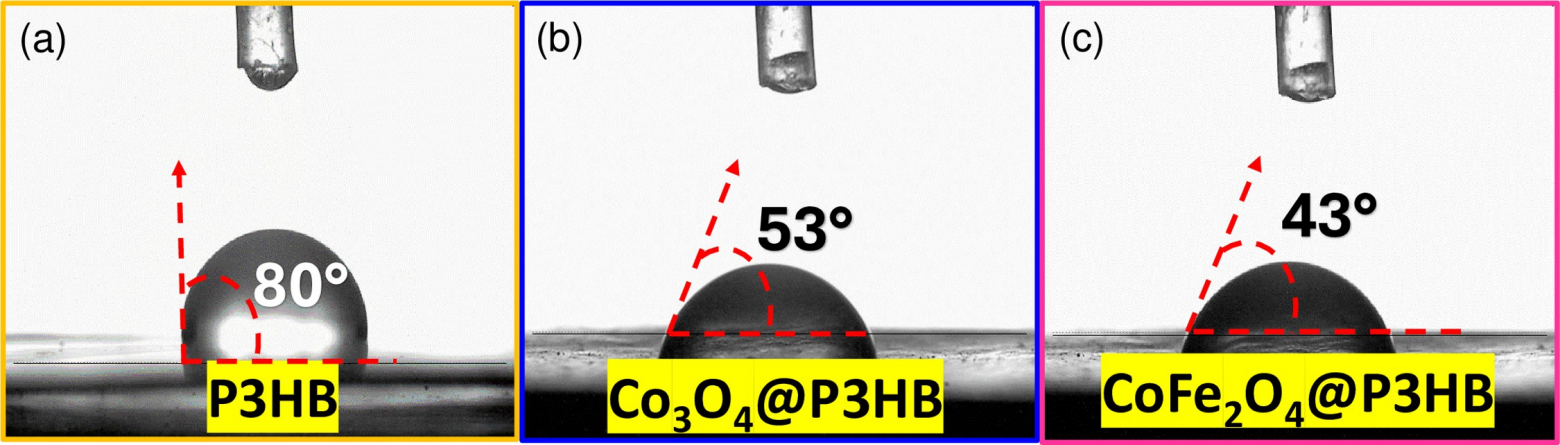
Representative photographs of the water droplet shape to measure the contact angle values. (a) P3HB film, (b) Co_3_O_4_@P3HB film and (c) CoFe_2_O_4_@P3HB film.

The hydrophilicity of the films was determined using the contact angle technique. Table 1 presents the average contact angle measurements for three films of each system. The contact angle for the P3HB film was 80° ± 2, while for the Co_3_O_4_@P3HB and CoFe_2_O_4_@P3HB hybrid films, it was 53° ± 2 and 43° ± 2, respectively. Representative photographs of the water droplet shape used for measuring the contact angles of the films are shown in Fig 16.

**Table 1:** Contact angles of P3HB film and hybrid films.

Contact Angle θ°		
Polymer	Hybrid films	
P3HB	Co_3_O_4_@P3HB	CoFe_2_O_4_@P3HB
**80 ± 2**	**53 ± 2**	**43 ± 2**

### Photodegradation studies

**Fig 17:**
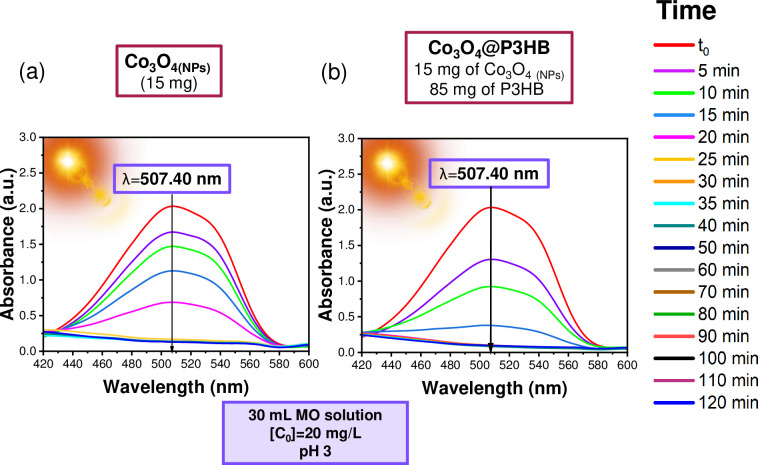
UV-Vis absorption spectra corresponding to the photodegradation of a solution of MO at pH 3, with an initial concentration (C_0_) of 20 mg/L, in a volume of 30 mL. In the presence of (a) Co_3_O_4_ NPs, (b) Co_3_O_4_@P3HB hybrid film. (a) and (b) Were exposed directly to sunlight.

**Fig 18:**
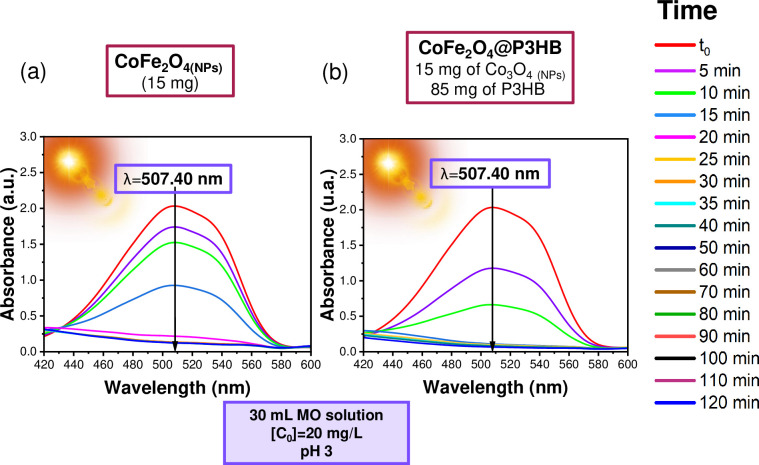
UV-Vis absorption spectra corresponding to the photodegradation of a solution of MO at pH 3, with an initial concentration (C_0_) of 20 mg/L, in a volume of 30 mL. In the presence of (a) CoFe_2_O_4_ NPs, (b) CoFe_2_O_4_@P3HB hybrid film. (a) and (b) Were exposed directly to sunlight.

The study of sunlight-induced degradation of methyl orange (MO) in its acid form was conducted, employing the Co_3_O_4_ and CoFe_2_O_4_ NPs and Co_3_O_4_@P3HB and CoFe_2_O_4_@P3HB hybrid films previously obtained. In Figs 17 and 18, the behavior of the absorption band at 507.40 nm over time is observed. This band is associated with MO’s quinoid structure (N-N-H) in its acidic form. Fig 17A and 17B show the photodegradation induced by the Co_3_O_4_ NPs and the Co_3_O_4_@P3HB hybrid film, while Fig 18A and 18B show the same for CoFe_2_O_4_ NPs and CoFe_2_O_4_@P3HB, respectively. Both systems successfully promote the photodegradation of MO when they are directly exposed to sunlight.

**Fig 19:**
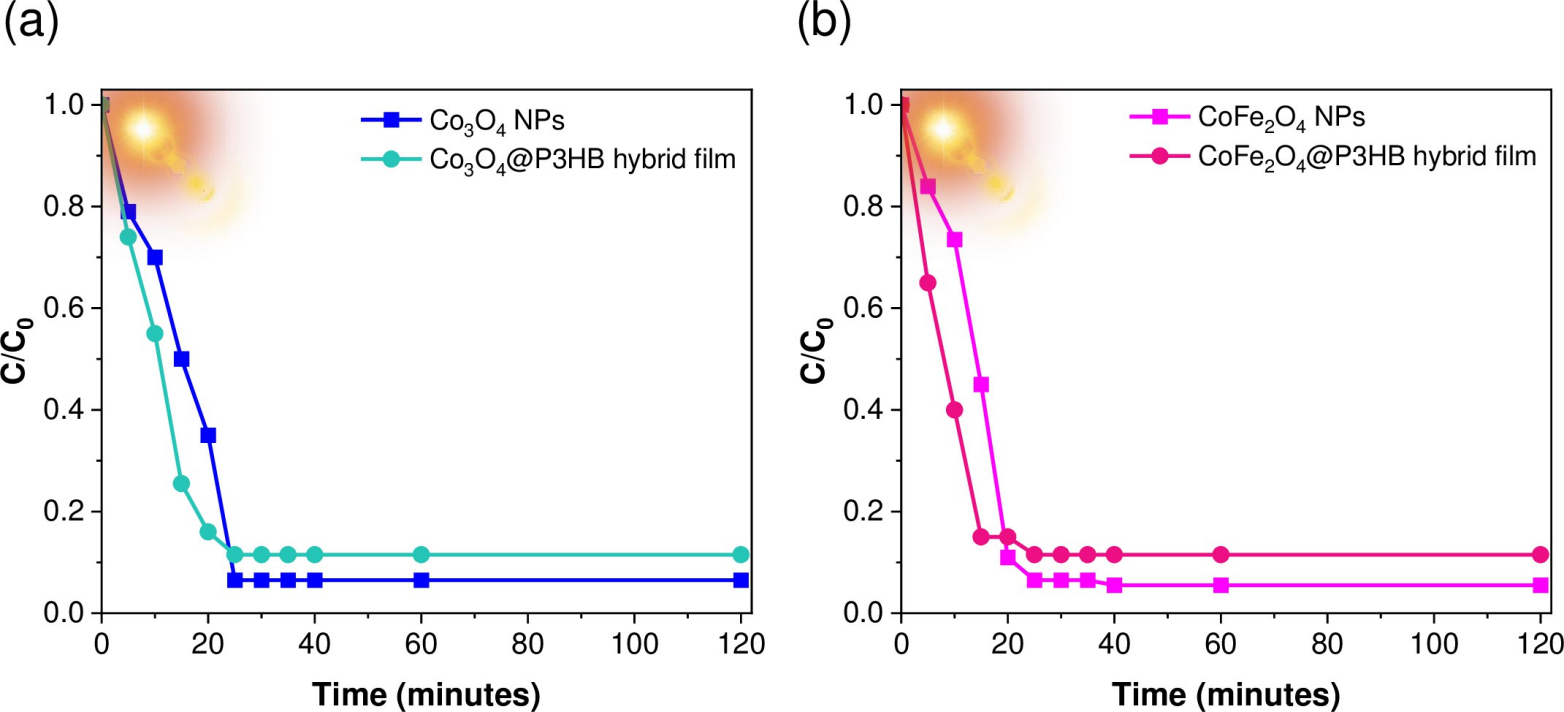
(C/C_0_) vs sunlight exposure time (min). (a) Co_3_O_4_ NPs and Co_3_O_4_@P3HB hybrid film. (b) CoFe_2_O_4_ NPs and CoFe_2_O_4_@P3HB hybrid film.

On the other hand, Fig 19 illustrates the change in MO concentration (C/C_0_) over time exposed to sunlight. Fig 19A displays the results for the Co_3_O_4_ NPs and Co_3_O_4_@P3HB film, while Fig 19B depicts the results obtained with CoFe_2_O_4_ NPs and CoFe_2_O_4_@P3HB film. In both cases, MO degradation was achieved in less than 30 minutes.

**Fig 20:**
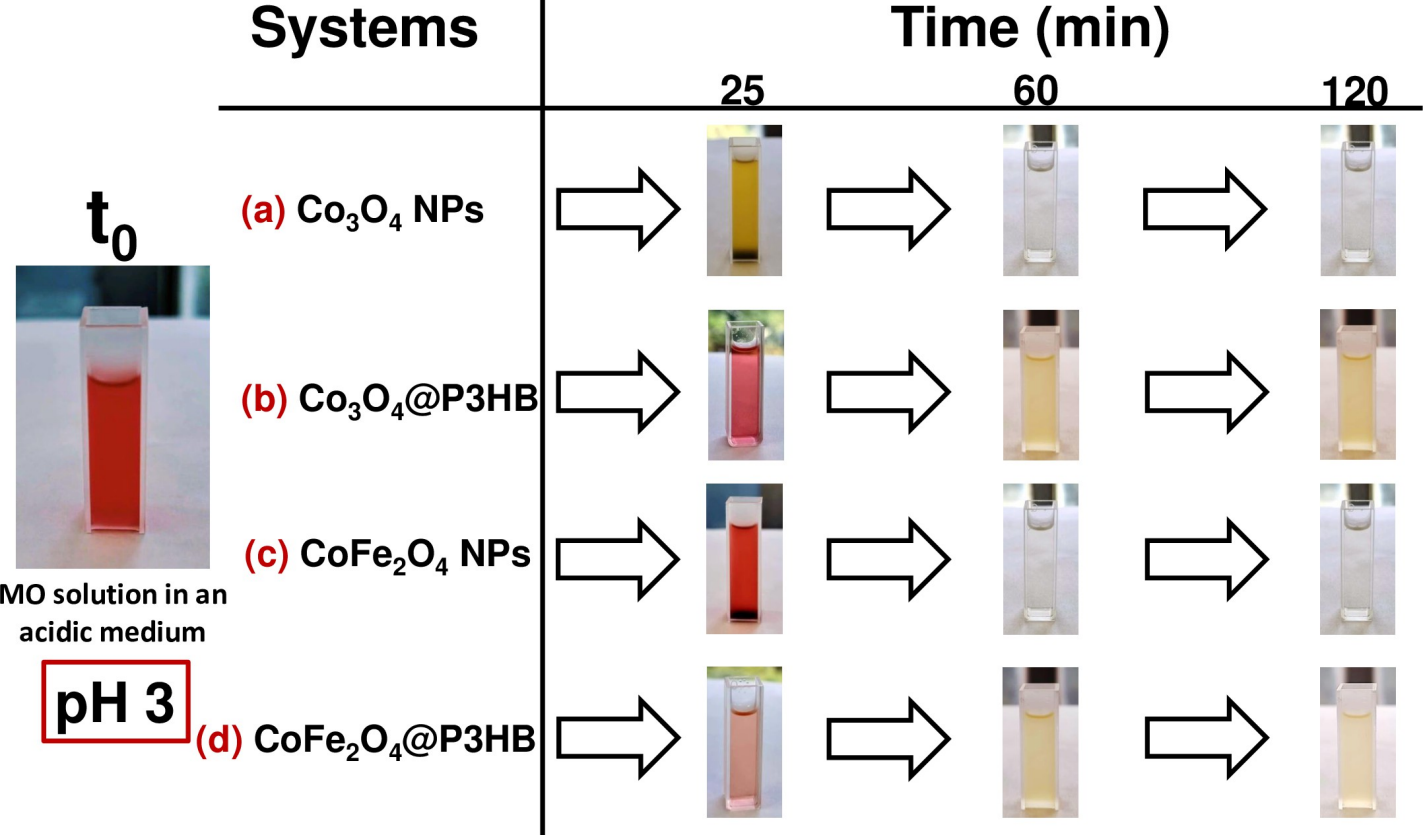
Representative images for the MO photodegradation process at different time intervals. (a) Co_3_O_4_ NPs, (b) Co_3_O_4_@P3HB hybrid film, (c) CoFe_2_O_4_ NPs, and (d) CoFe_2_O_4_@P3HB hybrid film.

Although better results were obtained with the isolated nanoparticles due to a larger surface contact area, incorporating the nanoparticles into a polymeric matrix offers broader potential applications. Fig 20 shows the colour changes observed in each system of nanoparticles and hybrid films over time.

In addition, the degradation efficiency of MO was evaluated using Eq (2). Table 2 presents the degradation efficiency (%) of methyl orange (MO) under sunlight exposure. The experiment involved 15 mg of Co_3_O_4_ or CoFe_2_O_4_ NPs dispersed in 30 mL of 20 mg/L of MO solution at pH 3. Additionally, the table contains data on the degradation efficiency under the same conditions for the Co_3_O_4_@P3HB and CoFe_2_O_4_@P3HB hybrid films obtained from 15 mg of nanoparticles dispersed in 85 mg of P3HB. All experiments were performed in triplicate.

**Table 2:** Photodegradation efficiency under sunlight illumination (%) vs Time (min).

	Photodegradation efficiency (%)			
	under sunlight illumination			
Time	Co_3_O_4_ NPs	Co_3_O_4_@P3HB	CoFe_2_O_4_ NPs	CoFe_2_O_4_@P3HB
minutes	(15mg)	(15 mg/85 mg)	(15 mg)	(15 mg/85 mg)
0	0	0	0	0
5	21 ± 1	26 ± 1	16 ± 1	35 ± 2
10	30 ± 2	45 ± 2	27 ± 1	60 ± 2
**15**	**50 ± 2**	**75 ± 2**	**55 ± 2**	**85 ± 2**
20	65 ± 2	84 ± 2	89 ± 2	89 ± 2
**25**	**94 ± 3**	**89 ± 3**	**94 ± 3**	**90 ± 2**
30	94 ± 3	89 ± 3	94 ± 3	92 ± 3
35	94 ± 3	89 ± 3	94 ± 3	92 ± 3
40	94 ± 3	89 ± 3	94 ± 3	92 ± 3
60	94 ± 3	89 ± 3	94 ± 3	92 ± 3
120	94± 3	89 ± 3	94 ± 3	92 ± 3

The results in Table 2 show that using isolated nanoparticles leads to 50% photodegradation efficiency for Co_3_O_4_ NPs and 55% for CoFe_2_O_4_ NPs after 15 minutes of sunlight exposure. However, the photodegradation significantly increased when the hybrid films were used under the same conditions, reaching 75% for the Co_3_O_4_@P3HB film and 85% for the CoFe_2_O_4_@P3HB film at 15 minutes. After 25 minutes of sunlight exposure, the photodegradation of the isolated nanoparticles and the hybrid films was very similar, reaching 94% and around 90%, respectively. After 30 minutes, no changes were observed in either system. These results represent a significant finding because the hybrid films have a lower concentration of nanoparticles in contact with the MO solution than the isolated nanoparticles, which have a larger contact area.

**Fig 21:**
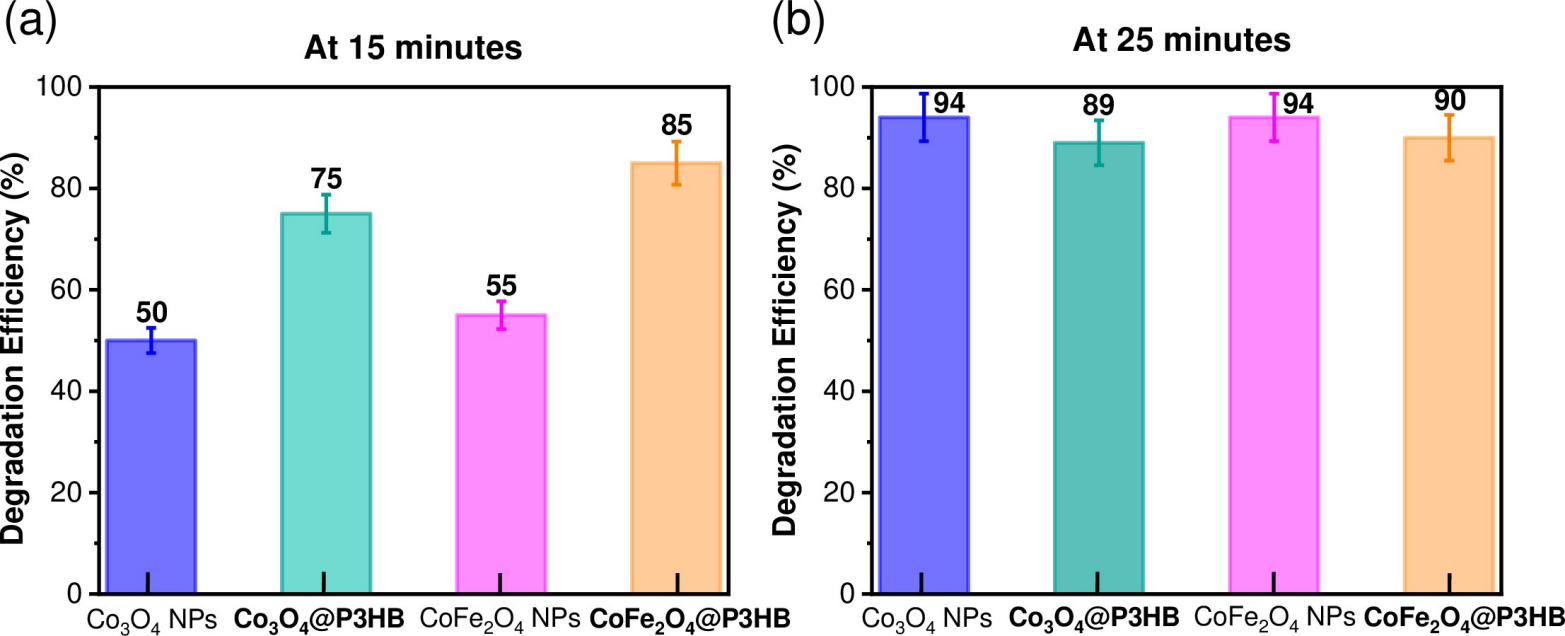
Photodegradation efficiency (%) of Co_3_O_4_ and CoFe_2_O_4_ nanoparticles, and Co_3_O_4_@P3HB and CoFe_2_O_4_@P3HB hybrid films after sunlight exposure (a) 15 minutes and (b) 25 minutes.

However, a valid comparison can only be made between NPs and hybrid films fabricated with NPs of the same chemical composition. In this sense, as shown in Fig 21A and 21B, the Co_3_O_4_@P3HB hybrid film was 25% more efficient than Co_3_O_4_ NPs, and the CoFe_2_O_4_@P3HB film was 35% more efficient than CoFe_2_O_4_ NPs after 25 minutes of exposure to sunlight. Besides, the higher hydrophilicity of the CoFe_2_O_4_@P3HB film could contribute to its improved initial response.

These results suggest that the P3HB polymer also acts synergistically with the NPs to promote the photodegradation of MO. This would explain why, over time, both NP systems and hybrid films reach similar photodegradation efficiencies. Under the same conditions evaluated, the P3HB film without nanoparticles showed a maximum photodegradation efficiency of 10% after 30 minutes of sunlight exposure and remained unchanged (not shown here).

**Fig 22:**
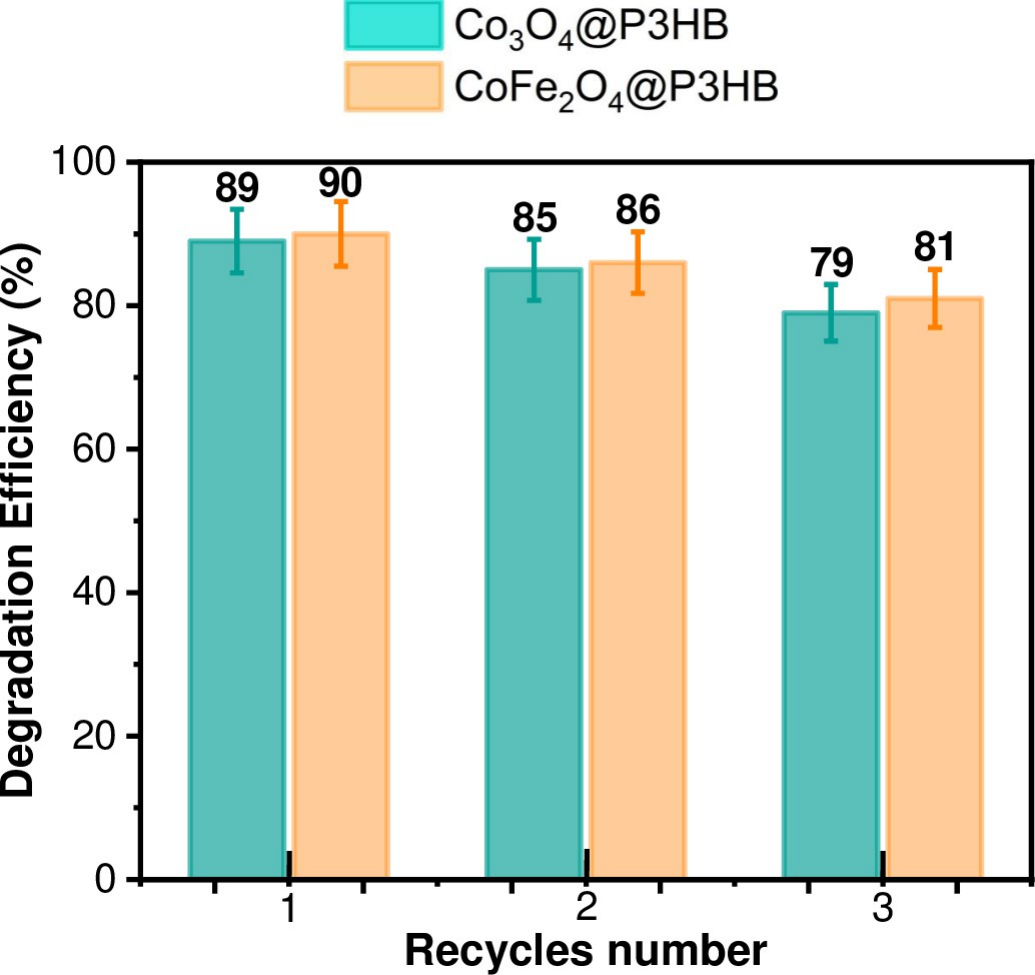
Photodegradation efficiency (%) for the Co_3_O_4_@P3HB and CoFe_2_O_4_@P3HB hybrid films after 25 minutes under sunlight exposure and three use cycles.

Furthermore, Fig 22 presents the photodegradation efficiency (%) of the MO by the hybrid films after 25 minutes of exposure to sunlight and three cycles of use. It was observed that the photodegradation efficiency decreased from 89% to 79% for the Co_3_O_4_@P3HB film and from 90% to 81% for the CoFe_2_O_4_@P3HB film after the third cycle of use. This decrease of around 10% in efficiency could be attributed to the loss of some active sites after each use.

**Fig 23:**
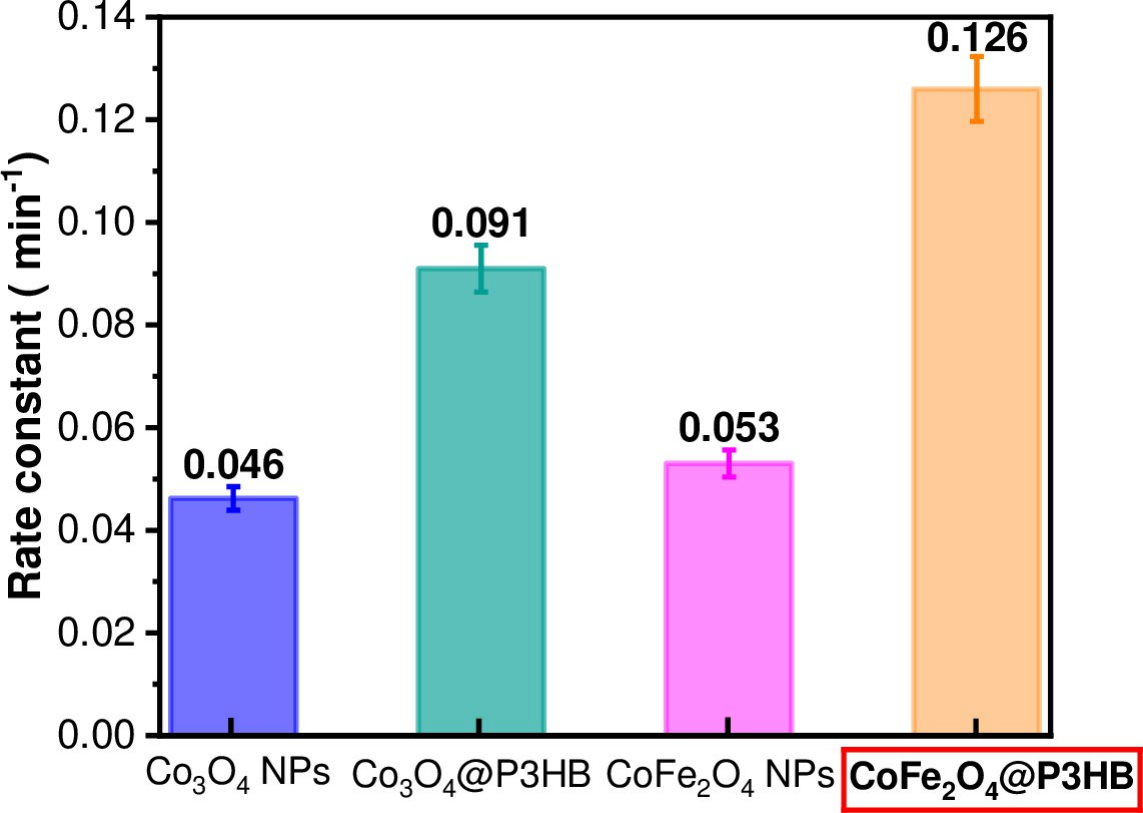
Photodegradation rate constants of the Co_3_O_4_ and CoFe_2_O_4_ NPs and Co_3_O_4_@P3HB and CoFe_2_O_4_@P3HB hybrid films after 15 minutes of sunlight exposure.

The photodegradation rate constants in min^-1^ for all the systems after 15 min of sunlight exposure **(**Fig 23**)** were estimated to be 0.046 ± 0.002 and 0.091 ± 0.004 for Co_3_O_4_ NPs and Co_3_O_4_@P3HB film. For CoFe_2_O_4_ NPs and CoFe_2_O_4_@P3HB film, 0.053 ± 0.003 and 0.126 ± 0.006, respectively. According to these results, the hybrid films show the highest rate of photodegradation. These findings are consistent with previous studies suggesting that the reaction rate of dye photodegradation can be explained by a model with pseudo-first-order kinetic rate constants [44].

The degradation of the methyl orange molecule in its acid form employing metal oxide nanoparticles (Co_3_O_4_ or CoFe_2_O_4_ NPs) incorporated in a polymeric matrix (P3HB) under solar light can be achieved through the following equations:

$$\;Hybrid\;films\;+hv\to e^{-}+h^{+}$$

$$h^{+}+H_{2}O\to HO•+H^{+}$$

$$e^{-}+O_{2}\to O_{2}$$

$$O_{2}+H_{2}O\to HO_{2}+OH^{-}$$

**Fig 24:**
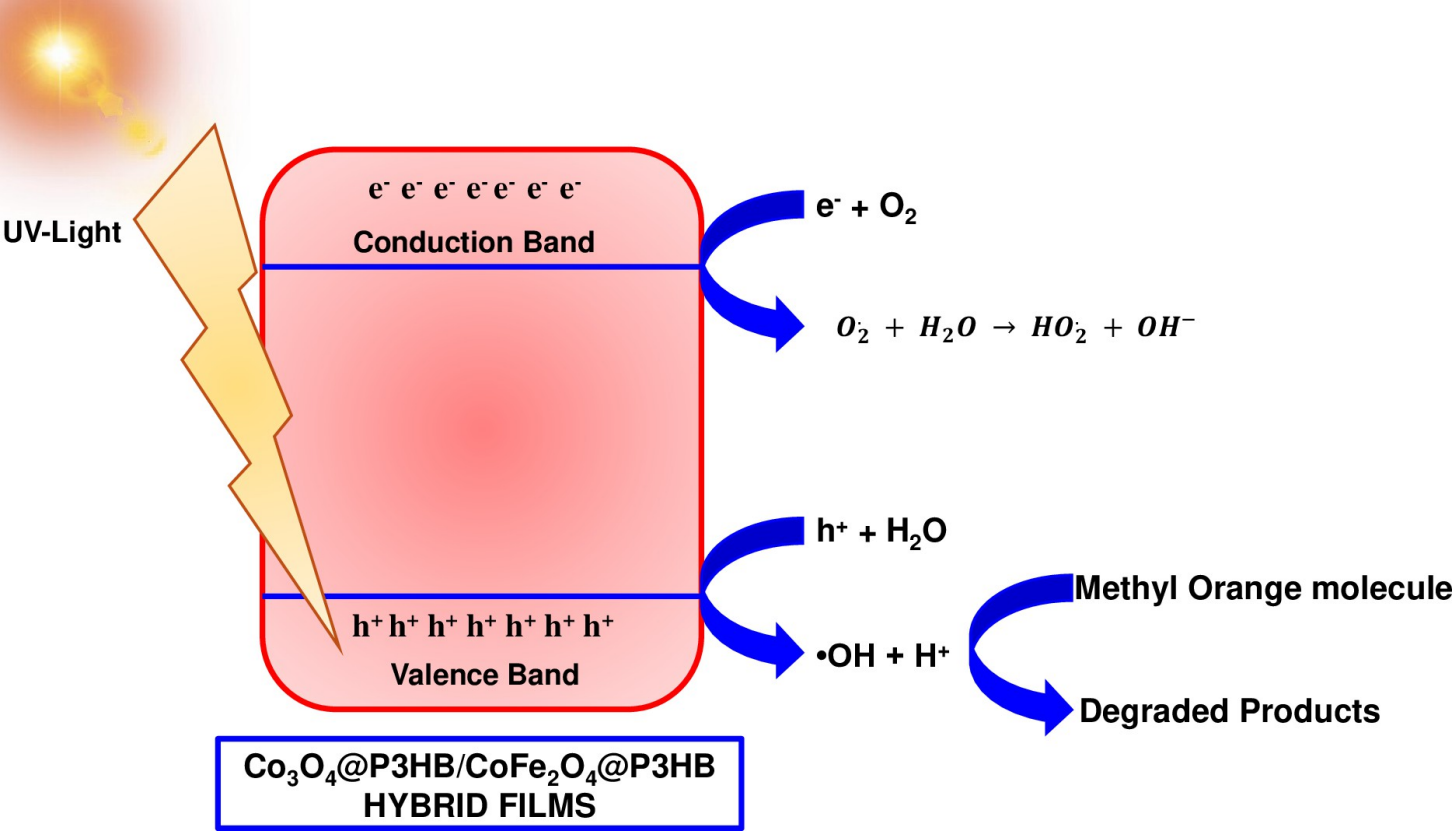
Schematic representation of the MO photodegradation mechanism by the Co_3_O_4_@P3HB and CoFe_2_O_4_@P3HB hybrid films under sunlight exposure.

This process (Fig 24) is made possible by the absorption of photons by the cobalt oxide or the cobalt ferrite on the surface of the hybrid films. These create holes in the valence band and excite the electrons in the conduction band. The holes then react with H_2_O to form •OH and H^+^. Meanwhile, the electrons in the conduction band react with dissolved oxygen to form superoxide radicals. The superoxide radicals can further react with H_2_O to form perhydroxyl radicals. Hydroxyl radicals, superoxide, and perhydroxyl compounds effectively degrade methyl orange in its acid form [44].

Finally, the CoFe_2_O_4_@P3HB hybrid film exhibits a ferrimagnetic behaviour at room temperature and a better photodegradation rate at 15 minutes, making it the most attractive material for future applications since it could be removed from a medium through a magnet. Future research might evaluate different concentrations of nanoparticles within the P3HB polymeric matrix to achieve 100% photodegradation of the MO. Furthermore, it could be extended to assess the response of such films to different pHs.

This work aimed to create two hybrid films using a biopolymer and nanoparticles of a mixed oxide of cobalt (II, III) and cobalt ferrite through a simple and reproducible method. This motivates future research to produce hybrid films with other polymers and with different metal oxide nanoparticles.

## Conclusions

The successful incorporation of Co_3_O_4_ and CoFe_2_O_4_ nanoparticles in a P3HB polymeric matrix has led to hybrid materials that significantly enhance the photodegradation of methyl orange in its acid form. Incorporating nanoparticles into the polymeric matrix increases the hydrophilicity and the roughness of the film surfaces, favouring the interaction with the MO solution. Photodegradation measurements have revealed that combining the nanoparticles with the polymer significantly improves the degradation performance compared to isolated nanoparticles. The CoFe_2_O_4_@P3HB film, in particular, has exhibited the best photodegradation performance, achieving a photodegradation efficiency of 85% in only 15 minutes of exposure to sunlight and a photodegradation rate of 0.126 min^-1^. These findings inspire the potential use of CoFe_2_O_4_@P3HB hybrid films in addressing various environmental challenges, given their promising biodegradability, magnetic response, cost-effectiveness, and high photodegradation activity.

## References

[1] Baig N, Kammakakam I, Falath W. Nanomaterials: A review of synthesis methods, properties, recent progress, and challenges. Mater. Adv. 2021;2(6):1821–1871.

[2] Abid N, Khan AM, Shujait S, Chaudhary K, Ikram M, Imran M, et al. Synthesis of nanomaterials using various top-down and bottom-up approaches, influencing factors, advantages, and disadvantages: A review. Adv. Colloid Interface Sci. 2022;300:102597. doi: 10.1016/j.cis.2021.102597 34979471

[3] Harish V, Tewari D, Gaur M, Yadav AB, Swaroop S, Bechelany M, et al. Review on nanoparticles and nanostructured materials: Bioimaging, biosensing, drug delivery, tissue engineering, antimicrobial, and agro-food applications. Nanomater. 2022;12(3):457. doi: 10.3390/nano12030457 35159802 PMC8839643

[4] Nicole L, Laberty-Robert C, Rozes L, Sanchez C. Hybrid materials science: a promised land for the integrative design of multifunctional materials. Nanoscale. 2014;6(12):6267–6292. doi: 10.1039/c4nr01788a 24866174

[5] Rejab MRBM, Hamdan MHBM, Quanjin M, Siregar JP, Bachtiar D, Muchlis Y. Historical development of hybrid materials. In: Encyclopedia of renewable and sustainable materials;2020. pp. 445–455.

[6] Adimule V, Bathula C, Sharma K, Keri R. Study of structural, optical, dielectric properties poly-3-butyl thiophene reinforced with Co_3_O_4_ nanoparticles. Nano-Struct. Nano-Objects. 2024;38:101150.

[7] Kickelbick G. Hybrid materials: synthesis, characterization, and applications: John Wiley & Sons;2007.

[8] Pandey V, Adiba A, Munjal S, Ahmad T. Optical bandgap tuning of cubic spinel Co_3_O_4_ by annealing temperature. Materialia. 2022;26:101554.

[9] Maiyalagan T, Jarvis KA, Therese S, Ferreira PJ, Manthiram A. Spinel-type lithium cobalt oxide as a bifunctional electrocatalyst for the oxygen evolution and oxygen reduction reactions. Nat. Commun. 2014;5(1):3949. doi: 10.1038/ncomms4949 24862287

[10] Waris A, Din M, Ali A, Afridi S, Baset A, Khan AU, et al. Green fabrication of Co and Co_3_O_4_ nanoparticles and their biomedical applications: A review. Open Life Sci. 2021;16(1):14–30. 33817294 10.1515/biol-2021-0003 PMC7968533

[11] Dehno Khalaji A, Jarosova M, Machek P, Chen K, Xue D. Co_3_O_4_ Nanoparticles: synthesis, characterization and its application as performing anode in Li-Ion batteries. J. Nanostruct. 2020;10(3):607–612.

[12] Reddy NR, Reddy PM, Mandal TK, Reddy KR, Shetti NP, Saleh TA, et al. Synthesis of novel Co_3_O_4_ nanocubes-NiO octahedral hybrids for electrochemical energy storage supercapacitors. J. Environ. Manage. 2021;298:113484. 34391101 10.1016/j.jenvman.2021.113484

[13] Yousefi SR, Alshamsi HA, Amiri O, Salavati-Niasari M. Synthesis, characterization and application of Co/Co_3_O_4_ nanocomposites as an effective photocatalyst for discoloration of organic dye contaminants in wastewater and antibacterial properties. J. Mol. Liq. 2021;337:116405.

[14] Hoilnsworth B, Mazumdar D, Sims H, Sun Q, Yurtisigi M, Sarkar S, et al. Chemical tuning of the optical band gap in spinel ferrites: CoFe_2_O_4_ vs NiFe_2_O_4_. Appl. Phys. Lett. 2013;103:082406.

[15] Agú UA, Oliva MI, Marchetti SG, Heredia AC, Casuscelli SG, Crivello ME. Synthesis and characterization of a mixture of CoFe_2_O_4_ and MgFe_2_O_4_ from layered double hydroxides: Band gap energy and magnetic responses. J. Magn. Magn. Mater. 2014;369:249–259.

[16] Kumar Prabhakar P, Vijayaraghavan S, Philip J, Doble M. Biocompatibility studies of functionalized CoFe_2_O_4_ magnetic nanoparticles. Curr. Nanosci. 2011;7(3):371–376.

[17] Lakshmi B, Thomas BJ, Gopinath P. Accurate band gap determination of chemically synthesized cobalt ferrite nanoparticles using diffuse reflectance spectroscopy. Adv. Powder Technol. 2021;32(10):3706–3716.

[18] Vazquez-Olmos AR, Abatal M, Sato-Berru RY, Pedraza-Basulto GK, Garcia-Vazquez V, Sainz-Vidal A, et al. Mechanosynthesis of MFe_2_O_4_ (M = Co, Ni, and Zn) magnetic nanoparticles for Pb removal from aqueous solution. J. Nanomater. 2016;2016:1–9.

[19] Mitra S, Srinivas P, Chakraborthy A, Petla RK. Electrochemical properties of spinel cobalt ferrite nanoparticles with sodium alginate as interactive binder. ChemElectroChem. 2014;1.

[20] Cruz DRS, Santos BTJ, Cunha GC, Romao LPC. Green synthesis of a magnetic hybrid adsorbent (CoFe_2_O_4_/NOM): Removal of chromium from industrial effluent and evaluation of the catalytic potential of recovered chromium ions. J. Hazard Mater. 2017;334:76–85. 28402897 10.1016/j.jhazmat.2017.03.062

[21] Simonescu CM, Tătăruş A, Culiţă DC, Stănică N, Ionescu IA, Butoi B, et al. Comparative study of CoFe_2_O_4_ nanoparticles and CoFe_2_O_4_-Chitosan composite for congo red and methyl orange removal by adsorption. Nanomater. 2021;11(3):711. 10.3390/nano11030711 PMC8001270 33808975

[22] Choudhary S, Bisht A, Mohapatra S. Facile synthesis, morphological, structural, photocatalytic and optical properties of CoFe_2_O_4_ nanostructures. SN Appl. Sci. 2019;1(12).

[23] Park J-W, Ju Y-W. Evaluation of Bi-Functional Electrochemical Catalytic Activity of Co_3_O_4_-CoFe_2_O_4_ composite spinel oxide. Energies. 2022;16(1):173.

[24] De Sousa Junior RR, Cezario FEM, Antonino LD, Dos Santos DJ, Lackner M. Characterization of Poly(3-hydroxybutyrate) (P3HB) from Alternative, Scalable (Waste) Feedstocks. Bioeng. 2023;10(12):1382. doi: 10.3390/bioengineering10121382 38135973 PMC10740857

[25] Yu J. Chapter 23—Microbial production of bioplastics from renewable resources. In: Yang S-T, editor. Bioprocessing for value-added products from renewable resources. Amsterdam: Elsevier; 2007. pp. 585–610.

[26] Keskin G, Kızıl G, Bechelany M, Pochat-Bohatier C, Öner M. Potential of polyhydroxyalkanoate (PHA) polymers family as substitutes of petroleum based polymers for packaging applications and solutions brought by their composites to form barrier materials. Pure and Appl. Chem. 2017;89(12):1841–1848.

[27] Popa MS, Frone AN, Panaitescu DM. Polyhydroxybutyrate blends: A solution for biodegradable packaging?. Int. J. Biol. Macromol. 2022;207:263–277. doi: 10.1016/j.ijbiomac.2022.02.185 35257732

[28] Raza ZA, Khalil S, Abid S. Recent progress in development and chemical modification of poly(hydroxybutyrate)-based blends for potential medical applications. Int. J. Biol. Macromol. 2020;160:77–100. doi: 10.1016/j.ijbiomac.2020.05.114 32439444

[29] Basnett P, Matharu RK, Taylor CS, Illangakoon U, Dawson JI, Kanczler JM, et al. Harnessing Polyhydroxyalkanoates and Pressurized Gyration for Hard and Soft Tissue Engineering. ACS Appl. Mater. Interfaces. 2021;13(28):32624–32639. doi: 10.1021/acsami.0c19689 34228435

[30] Akaraonye E, Filip J, Safarikova M, Salih V, Keshavarz T, Knowles JC, et al. P(3HB) Based Magnetic Nanocomposites: Smart Materials for Bone Tissue Engineering. J. Nanomater. 2016;2016:1–14.

[31] Hayashi K, Maeda K, Moriya M, Sakamoto W, Yogo T. In situ synthesis of cobalt ferrite nanoparticle/polymer hybrid from a mixed Fe–Co methacrylate for magnetic hyperthermia. J. Magn. Mag. Mater. 2012;324(19):3158–3164.

[32] Arun T, Verma SK, Panda PK, Joseyphus RJ, Jha E, Akbari-Fakhrabadi A, et al. Facile synthesized novel hybrid graphene oxide/cobalt ferrite magnetic nanoparticles based surface coating material inhibit bacterial secretion pathway for antibacterial effect. Mater. Sci. Eng. C. 2019;104:109932. doi: 10.1016/j.msec.2019.109932 31499934

[33] Ali N, Bilal M, Khan A, Ali F, Yang Y, Khan M, et al. Dynamics of oil-water interface demulsification using multifunctional magnetic hybrid and assembly materials. J. Mol. Liq. 2020;312:113434.

[34] Lizundia E, Rincón-Iglesias M, Lanceros-Méndez S. Combining cobalt ferrite and graphite with cellulose nanocrystals for magnetically active and electrically conducting mesoporous nanohybrids. Carbohydr. Polym. 2020;236:116001. doi: 10.1016/j.carbpol.2020.116001 32172835

[35] Tung DT, Tam LTT, Dung HT, Dung NT, Ha HT, Dung NT, et al. Direct Ink writing of graphene–cobalt ferrite hybrid nanomaterial for supercapacitor electrodes. J. Electron. Mater. 2020;49(8):4671–4679.

[36] Pryadko A, Surmeneva MA, Surmenev RA. Review of hybrid materials based on polyhydroxyalkanoates for tissue engineering applications. Polym. 2021;13(11):1738. doi: 10.3390/polym13111738 34073335 PMC8199458

[37] Rincon-Granados KL, Vázquez-Olmos AR, Rodríguez-Hernández A-P, Prado-Prone G, Garibay-Febles V, Almanza-Arjona YC, et al. Bactericidal and cytotoxic study of hybrid films based on NiO and NiFe_2_O_4_ nanoparticles in poly-3-hydroxybutyrate. J. Clust. Sci. 2024;35(1):167–178. 10.3390/ijms242317052 PMC10707088 38069375

[38] Belardja MS, Dahou FZ, Djelad H, Benyoucef A. Adsorption and supercapacitor applications of CoO-zeolite decorated conducting polymer nanocomposite. Intern. J. Environ. Anal. Chem. 2024:1–14.

[39] Waghchaure RH, Adole VA, Jagdale BS. Photocatalytic degradation of methylene blue, rhodamine B, methyl orange and Eriochrome black T dyes by modified ZnO nanocatalysts: A concise review. Inorg. Chem. Commun. 2022;143:109764.

[40] Wu L, Liu X, Lv G, Zhu R, Tian L, Liu M, et al. Study on the adsorption properties of methyl orange by natural one-dimensional nano-mineral materials with different structures. Sci. Rep. 2021;11(1):10640. doi: 10.1038/s41598-021-90235-1 34017049 PMC8138017

[41] Azad K, Gajanan P. Photodegradation of methyl orange in aqueous solution by the Visible Light Active Co:La:TiO_2_ nanocomposite. Chem. Sci. J. 2017;8(3).1000164–1000174.

[42] Iwuozor KO, Ighalo JO, Emenike EC, Ogunfowora LA, Igwegbe CA. Adsorption of methyl orange: A review on adsorbent performance. Curr. Res. Green Sustain. Chem. 2021;4:100179.

[43] Ter-Zakaryan A, Zhukov A. Materials horizons: From nature to nanomaterials. In: Materials Horizons: From Nature to Nanomaterials, editor. Springer; 2021. pp. 349–377.

[44] Aziztyana AP, Wardhani S, Prananto YP, Purwonugroho D, Darjito. Optimisation of methyl orange photodegradation using TiO_2_-zeolite photocatalyst and H_2_O_2_ in acid condition. IOP Conf. Ser. Mater. Sci. Eng. 2019;546(4):042047.

[45] Sun J, Wang H, Li Y, Zhao M. Porous Co_3_O_4_ column as a high-performance Lithium anode material. J. Porous Mater. 2021;28(3):889–894.

[46] Srinivas M. Superior photocatalytic activity of Mn-doped CoFe_2_O_4_ under visible light irradiation: Exploration of hopping and polaron formation in the spinel structure. Mater. Sci. Eng. B. 2021;270:115222.

[47] Wang Y, Wei X, Hu X, Zhou W, Zhao Y. Effect of formic acid treatment on the structure and catalytic activity of Co_3_O_4_ for N_2_O decomposition. Catal. Lett. 2019;149(4):1026–1036.

[48] Yadav RS, Kuřitka I, Vilcakova J, Havlica J, Masilko J, Kalina L, et al. Impact of grain size and structural changes on magnetic, dielectric, electrical, impedance and modulus spectroscopic characteristics of CoFe_2_O_4_ nanoparticles synthesized by honey mediated sol-gel combustion method. Adv. Nat. Sci.: Nanosci. Nanotechnol. 2017;8(4):045002.

[49] Prabaharan DDM, Sadaiyandi K, Mahendran M, Sagadevan S. Precipitation method and characterization of cobalt oxide nanoparticles. Appl. Phys. A. 2017;123(4):1–6.

[50] Alzoubi GM, Albiss BA, Shatnawi M, Bsoul I, Alsmadi AM, Salameh B, et al. Influence of high-temperature annealing on structural and magnetic properties of crystalline cobalt ferrite nanoparticles in the single-domain regime. J. Superc. Novel Magn. 2020;33(10):3179–3188.

[51] Kalam A, Al-Sehemi AG, Assiri M, Du G, Ahmad T, Ahmad I, et al. Modified solvothermal synthesis of cobalt ferrite (CoFe_2_O_4_) magnetic nanoparticles photocatalysts for degradation of methylene blue with H_2_O_2_/visible light. Results Phys. 2018;8:1046–1053.

[52] Rajendran M, Pullar R, Bhattacharya A, Das D, Chintalapudi S, Majumdar C. Magnetic properties of nanocrystalline CoFe_2_O_4_ powders prepared at room temperature: variation with crystallite size. J. Magn. Magn. Mater. 2001;232(1–2):71–83.

