# Correction: Multifunctional hybrid films made from CoT_3_OT_x4_ and CoFeT_2_OT_4_ nanoparticles inside a poly 3-hydroxybutyrate matrix and study of their impact in methyl orange photodegradation

**DOI:** 10.1371/journal.pone.0320895

**Published:** 2025-03-25

**Authors:** Lan J. Bernal-Sánchez, América R. Vázquez-Olmos, Roberto Y. Sato-Berrú, Esther Mata-Zamora, Margarita Rivera, Vicente Garibay-Febles

The chemical formulas in the article title is incorrect. The correct title is: Multifunctional hybrid films made from Co_3_O_4_ and CoFe_2_O_4_ nanoparticles inside a poly 3-hydroxybutyrate matrix and study of their impact in methyl orange photodegradation. The correct citation is: Bernal-Sánchez LJ, Vázquez-Olmos AR, Sato-Berrú RY, Mata-Zamora E, Rivera M, Garibay-Febles V (2024) Multifunctional hybrid films made from CoT_3_OT_x4_ and CoFeT_2_OT_4_ nanoparticles inside a poly 3-hydroxybutyrate matrix and study of their impact in methyl orange photodegradation. PLoS ONE 19(10): e0312611. https://doi.org/10.1371/journal.pone.0312611.
